# Synthesis and characterization of Cu(OH)_2_-NWs-PVA-AC Nano-composite and its use as an efficient adsorbent for removal of methylene blue

**DOI:** 10.1038/s41598-021-84797-3

**Published:** 2021-03-11

**Authors:** Sivarama Krishna Lakkaboyana, Khantong Soontarapa, Nabel Kalel Asmel, Vinay Kumar, Ravi Kumar Marella, Ali Yuzir, Wan Zuhairi Wan Yaacob

**Affiliations:** 1 Department of Chemical Technology, Faculty of Sciences, Chulalongkorn University, Pathumwan, Bangkok, 10330 Thailand; 2 Center of Excellence on Petrochemical and Materials Technology, Chulalongkorn University, Pathumwan, Bangkok, 10330 Thailand; 3 Building and Construction Technology Engineering, Northern Technical University, 41002 Mosul, Iraq; 4 Department of Biotechnology, Indian Institute of Technology Roorkee, Roorkee, Uttarakhand 247667 India; 5 Department of Chemistry (H & S), PACE Institute of Technology & Sciences, Ongole, Andhra Pradesh 523001 India; 6 Department of Environmental Engineering and Green Technology (EGT), MJIIT- Universiti Teknologi Malaysia, Jalan Sultan Yahya Petra, 54100 Kuala Lumpur, Malaysia; 7 Geology Program, School of Environmental Science and Natural Resources, FST, University Kebangsaan Malaysia, 43600 Bangi, Selangor Malaysia

**Keywords:** Pollution remediation, Nanowires

## Abstract

The present study focused on the synthesis of copper hydroxide nanowires decorated on activated carbon (Cu(OH)_2_-NWs-PVA-AC). The obtained Cu(OH)_2_-NWs-PVA-AC Nano-composite was distinguished by XRD, SEM, EDX, BET, FTIR and XPS respectively. Besides, different variables such as solution p^H^, and initial dye concentration, contact time, and temperature were performed on the adsorption efficiency of MB in a small batch reactor. Further, the experimental results are analyzed by various kinetic models via PFO, PSO, intra-particle diffusion and Elovich models, and the results revealed that among the kinetic models, PSO shows more suitability. In addition, different adsorption isotherms were applied to the obtained experimental data and found that Langmuir–Freundlich and Langmuir isotherm were best fits with the maximum adsorption capacity of 139.9 and 107.6 mg/g, respectively. The Nano-composite has outstanding MB removal efficiency of 94–98.5% with a span of 10 min. and decent adsorption of about 98.5% at a p^H^ of 10. Thermodynamic constants like Gibbs free energy, entropy, and enthalpy were analyzed from the temperature reliance. The results reveal the adsorption processes are spontaneous and exothermic in nature. The high negative value of ΔG° (− 44.11 to − 48.86 kJ/mol) and a low negative value of ΔH° (− 28.96 kJ/mol) show the feasibility and exothermic nature of the adsorption process. The synthesized dye was found to be an efficient adsorbent for the potential removal of cationic dye (methylene blue) from wastewater within a short time.

## Introduction

Currently, dyes are a common pollutant in the modernized society; dyes are hugely used in textiles, dyeing, printing, tanneries, electroplating, and associated industries^1,2^. These industries produce a large amount of polluted wastewater which is expelled into the water bodies and in turn affects the environment. Synthetic dyes are well-known as one of the major environmental toxic and anthropogenic which typically cause severe deterioration to plants and organisms in the ecosystem^3,4^. A study showed that treating the dyes merely is a difficult process because they are non-biodegradable, besides, the molecular structure of dyes has a very complex aromatic compound which makes dyes in water more stable^5^. Thus, the elimination of dyes from industrial wastewater is a difficult task for contemporary researchers. However, once they are successfully extracted it goes a long way to produce safe and purified water. Even the most reduced dose of dyes might affect aquatic life, and it causes the penetration of light and may cause disorder in the ecosystem^3–5^ MB dye consists of several applications in various fields such as chemistry, medical science, biology, and dyeing industries. Its continuous exposure can cause hypertension, vomiting, anemia, and nausea^6–8^. Currently, the development of sustainable and green synthetic methods using nanoparticles is considered a significant challenge for researchers. Therefore, more research studies should focus on the development of eco-friendly and efficient treatment method^9^. Copper nano-composites are drawn huge attention in the recent decade due to their outstanding properties that enable their usage in countless applications in variable fields, including structural materials, electronics, and adsorption^10^. Different methods were used to eliminate the dyes from contaminated water, such as chemical, physical, and biological processes, mainly adsorption (chemical and biosorption), coagulation/flocculation, ozonation, oxidation, liquid–liquid extraction, and membrane filtration^9–11^. The benefits and drawbacks of every technique have been broadly reviewed in the review articles. Among all techniques, adsorption represents a comparatively sustainable process because of the ease of operation, exceptional capacity, efficiency, and large-scale ability of regeneration to adsorbents. Therefore, most of the researchers suggest adsorption for wastewater treatment^11–13^. Traditionally, nanomaterial and Nanocomposite adsorbents have notable chemical and physical properties such as high surface area, reactive surface sites with more pore volume^14^. Consequently, nowadays contemporary researchers much emphasize using nanomaterial and Nanocomposite adsorbents in adsorption and separation processes^15^. Nano-composite materials have excellent adsorption and desorption properties for pollutants. These hybrid Nano-composite materials are easy to use and eco-friendly for removing the pollutants from the water^16,17^.

Activated carbon (AC) is the more productive adsorbent utilized in the dye removal process. However, it includes certain limitations like it’s expensive to produce and regenerate^13^. AC is low cost-effective and includes porous morphology with good and re-usable support for loading Nano-materials. At the same AC leads to an enhancement in distinct types of reactive spots and active points and also prolongs the lifetime of adsorbents, decreasing the toxicity in the separation processes^18,19^. Some metals and metal oxide nanoparticles are exceptionally dangerous to the aquatic environment^20^. Because of aforesaid limitations, we synthesized less toxic Nano-composites. In this case, the Nano-composite metal has been synthesized in the hydroxide form. In most cases, hydroxide forms of metals are very less toxic as compared to nanoparticles.

The present study demonstrates, synthesis and characterization of a new adsorbent, Cu(OH)_2_-NWs-PVA-AC Nano-composite. This Nano-composite adsorbent was applied for removal of MB and the kinetics and equilibrium of the sorption process were investigated and experimental data were analyzed to explore the isotherm of the adsorption process, rate of adsorption, and mechanisms.

## Materials and methods

### Materials

Activated carbon was procured from Pacoal Manufacturing Industry Sdn Bhd, Malaysia. PVA (Polyvinyl alcohol), CuNO_3_, Hydrochloric acid (HCl), Ammonium hydroxide (NH_4_OH), Sodium hydroxide (NaOH) used in the experiments were procured from Merck (Darmstadt, Germany). All the aqueous solutions are prepared by using Ultra-Pure Millipore Water (UPMW) of 18.2 MΩ cm (Millipore Corporation, USA). Methylene blue (C_16_H_18_N_3_SCl.3H_2_O) was procured from E. Merck, Thailand. All the chemical compounds used in the present study are analytical grade with high purity.

### Adsorbent preparation

#### Synthesis of Cu (OH)_2_ nanowires [Cu (OH)_2_ NWs]

The Cu(OH)_2_NWs was synthesized by following the chemical precipitation method^5^. Typically, 0.1 M Cu (NO_3_)_2_·3H_2_O in 100 mL Millipore water was kept under continuous stirring at 50 °C to produce a homogenous solution. Subsequently, NH_4_OH(aq) was added slowly dropwise to the Cu^2+^(aq) precursor, and the temperature raised to 80 °C, under controlled p^H^ conditions with continuous magnetic stirring to produce [Cu (NH_3_)_4_]^2+^ complex. During the reaction period, the p^H^ of the solution was controlled below 10 using sodium hydroxide. The [Cu(NH_3_)_4_]^2+^ complex changes into the Cu(OH)_2_NWs as a precipitate (ppt) as the excess of OH^−^ ions in the reactant mixture. Further, the resulting ppt was filtered carefully, washed with DI water and ethanol absolute for the complete removal of impurities. Finally, the solid product was dried properly at room temperature (~ 30 °C) for 3 h to get pure Cu(OH)_2_ NWs.

#### Synthesis of Cu(OH)_2_-NWs-PVA-AC

Approximately 4 g of PVA was mixed in 200 mL DI water and continuously stirred at 65 °C for 4 h to produce a homogenous solution of PVA. Considerably, 10 g of activated carbon was mixed into the PVA solution and stirred (100 rpm) for 30 min. Subsequently, 4 g of Cu(OH)_2_-NWs was mixed with the AC-PVA solution and the mixture was constantly stirred for 2 h to produce a homogenous suspension. After that, some amount of NH_4_OH was included dropwise to the mixture until the precipitate is developed. The composite mixture was stirred continuously at 60 °C for another 1 h, filtrated and washed with excess amount of heated DI water. The resulting Cu(OH)_2_-NWs-PVA-AC composite was dried at 60 °C for 12 h, powdered and kept in a desiccator for further use.

### Adsorbent characterization

Investigation of amorphous or crystalline phases and the particle size of Cu(OH)_2_-NWs and Cu(OH)_2_-NWs-PVA-AC was measured by powder XRD analysis. The XRD analysis was performed on a D8 ADVANCE (M/s. Bruker, Germany) X-ray diffractometer with Cu Kα radiation (λ = 1.54056 Å) operated at 40 kV and 30 mA. The diffraction lines are recorded in the 2θ values ranging from 20° to 80° with an increment of 0.02° per second and a scan speed of 1° per minute. The diffraction lines and the crystalline phases of diffractograms were compared with standard references informed in the JCPDS data file. The size of Cu(OH)_2_-NWs crystallites was measured from the FWHM value by adopting the Scherrer formula. FT-IR patterns of the adsorbent samples were documented on a Nicolet 6700 Spectrometer (M/s. Thermo Fisher Scientific, USA) within the wavenumber range of 4000–600 cm^−1^.

The X-ray photoelectron spectroscopy measurements were measured on an Axis 165 (M/s. Kratos Analytical Ltd., UK) XPS Spectrometer operated with Mg Kα radiation (1253.6 eV) over the adsorbent samples. The binding energy (B.E.) values of all the elements are corrected by using the C 1s (sp^3^ C, 284.6 eV) as a standard reference. The shape and complex morphology of the adsorbent samples are explored by the SEM measurements using Hitachi S-4800 (Hitachi High-Tech, Japan) under an acceleration voltage of 15 kV, and the elementary composition is typically obtained by energy-dispersive X-ray (EDX) analysis. The size distribution of Cu(OH)_2_-NWs and Cu(OH)_2_-NWs-PVA-ACwere examined by transmission electron microscopy.

### Adsorption experiments

Initially, we designated MB dye to test the adsorption ability of Cu(OH)_2_-NWs-PVA-AC. The adsorption test was typically performed at different p^H^ for the specific dye MB. A series of adsorption studies were conducted using 25 mL of MB solution in 200 mL Erlenmeyer flasks containing with 30 mg of Cu(OH)_2_-NWs-PVA-AC Nano-composite. The effect of p^H^ on the 50 mg/L of MB concentration was performed in the p^H^ range of 2‒10. The adsorption experiments are performed on a horizontal rotating water bath shaker with a shaking speed of 200 rpm. The effect of contact time was performed at different MB dye concentrations at 10‒30 mg/L and 0–60 min, respectively. Adsorption equilibrium isotherms were obtained with the different initial concentrations of MB (60‒100 mg/g) at various temperatures (35, 45, and 55 °C). These flasks were shaken continuously for 1 h with 200 rpm. Once the adsorption equilibrium is attained, the adsorbent and reacted dyes solution was extracted by centrifugation. Afterward, the dye concentration was calculated using the UV–Vis spectrophotometer by absorption at 668 nm. Triplicates were maintained for all the controlled experiments to check the repeatability and average values were provided. The quantitative amount of MB on the adsorbent (q_e_, mg/g) and removal efficiency (R) were estimated at the equilibrium conditions from the following Eqs. (1) and (2), respectively. The amount, q_t_ (mg/g), of MB adsorbed by Cu (OH)_2_-NW-PVA-AC at time ‘t’ and was calculated using Eq. (1).

1 $$q_{t} = \frac{{\left[ {\left( {C_{o}-C_{t} } \right) \cdot V} \right]}}{W}.$$

In Eq. (1), ‘C_o_’ and ‘C_t_’(mg/L) are the initial concentration and concentration at a time ‘t’, respectively. V (L) is the volume of MB solution, and W (mg) is the weight of the used Cu (OH)_2_-NW@AC. The MB elimination percentage onto Cu (OH)_2_-NW@AC was calculated using Eq. (2).

2 $$\% {\text{ Removal}} = \frac{{\left( {C_{o}-C_{e} } \right)}}{{C_{o} }} \times 100,$$

where C_e_ (mg/L) is the concentration of MB in solution at equilibrium.

### Statistical analysis

The experimental data was employed to various models by non-linear regression adapting to the standard method of least squares and curve fitting and statistical analyses were gained by Excel-Solver software^5,21^. The correlation coefficient (R^2^), residual root mean square error (RMSE), chi-square test (χ^2^), and standard error of the estimate (SE) were applied for the statistical analysis of the isotherm while the normalized standard deviation (NSD) and average relative error (ARE) were properly used for comparing direct applicability of the kinetics model achieved from non-linear model regressions.

### Ethics declarations

The present research does not include any human or animal subjects.

## Results and discussions

### Characterization of Cu(OH)_2_-NWs and Cu(OH)_2_-NWs-PVA-AC

**Figure 1:**
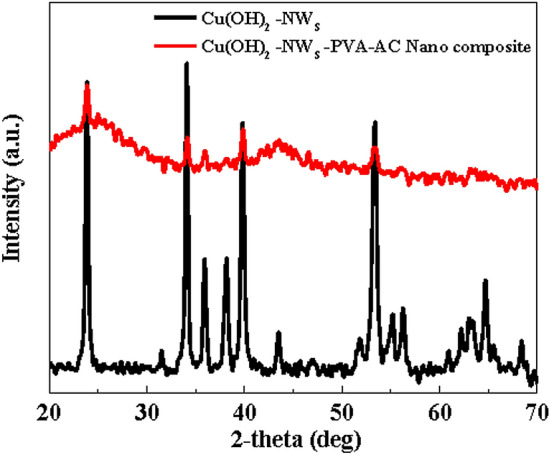
XRD patterns of Cu(OH)_2_-NWs, and Cu(OH)_2_-NWs-PVA-AC Nano-composite.

The X-ray diffraction (XRD) patterns of pure Cu(OH)_2_NWs and Cu(OH)_2_-NWs-PVA-AC is illustrated in Fig. 1. The well-resolved diffractions observed at 34.062°, 38.084°, 53.287°, 73.528°, and 75.87° corresponding to the lattice planes of (002), (022), (132), (202), and (222) respectively. The XRD reflections of both the samples are promptly confirming the active presence of Cu(OH)_2_ NWs (JCPDS No. 00-035-0505). The average crystallites size (D) is determined through the Debye–Scherrer equation based on the most intense peaks and were estimated to be around 22.68 nm, 27.52 nm for Cu(OH)_2_-NWs and Cu(OH)_2_-NWs-PVA-AC respectively. The broad diffraction peak at 2θ value from 15° to 30° is due to the amorphous carbon. The weak and broad reflection at 2θ value from 40° to 50° can be ascribed to the graphitic carbon. All these results confirmed the fabrication of the uniform Cu (OH)_2_-NWs on activated carbon. Simultaneously, the XRD patterns of Cu(OH)_2_-NWs-PVA-AC Nano-composite after MB dye adsorption are represented in Fig. S1 (Supplementary Information). On comparison, the position of XRD patterns did not alter significantly, which specifies that MB dye provides no influence on the crystalline structure of Cu(OH)_2_-NWs-PVA-AC Nano-composite.

**Figure 2:**
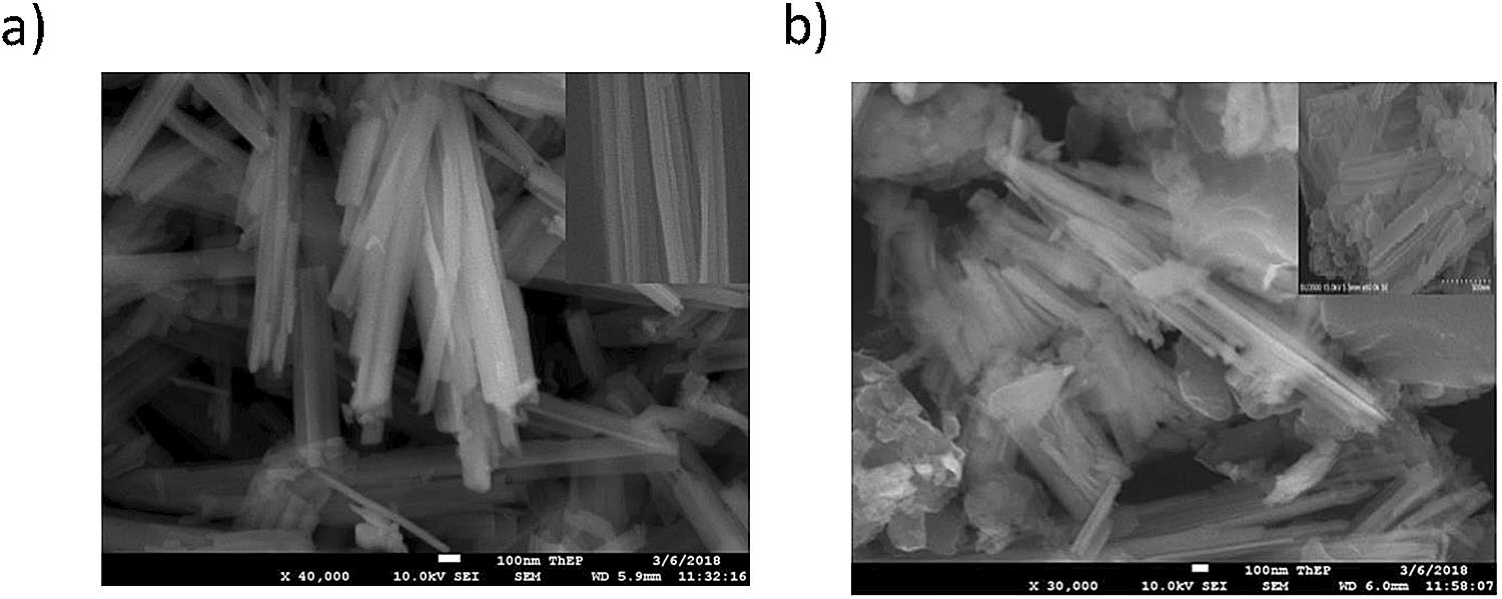
SEM images of (**a**) Cu(OH)_2_-NWs, and (**b**) Cu(OH)_2_-NWs-PVA-AC Nano-composite.

The SEM micrographs of the Cu(OH)_2_-NWs and the Cu(OH)_2_-NWs accumulated on activated carbon as shown in Fig. 2a,b. It can be noticed from Fig. 2a, the synthesized copper hydroxide exhibits a nanowire like morphology. These nanowires are aligned with straight and uniform size due to the surface regulation, oriented attachment, and strong van der Waals forces between the individual NWs. The morphology of Cu(OH)_2_-NWs-PVA-AC Nano-composite was shown in Fig. 2b, which indicate a large quantity of Cu(OH)_2_-NWs as bundles of several irregularly well-dispersed and composed on the activated carbon^22^. The FE-SEM image of Cu(OH)_2_-NWs-PVA-AC Nano-composite after adsorption of MB dye was depicted in Fig. S2 (Supplementary Information). It can be observed that uniform coverage of dye molecules on the exterior surface of the Nano-composite indicating its superior adsorption activity.

**Figure 3:**
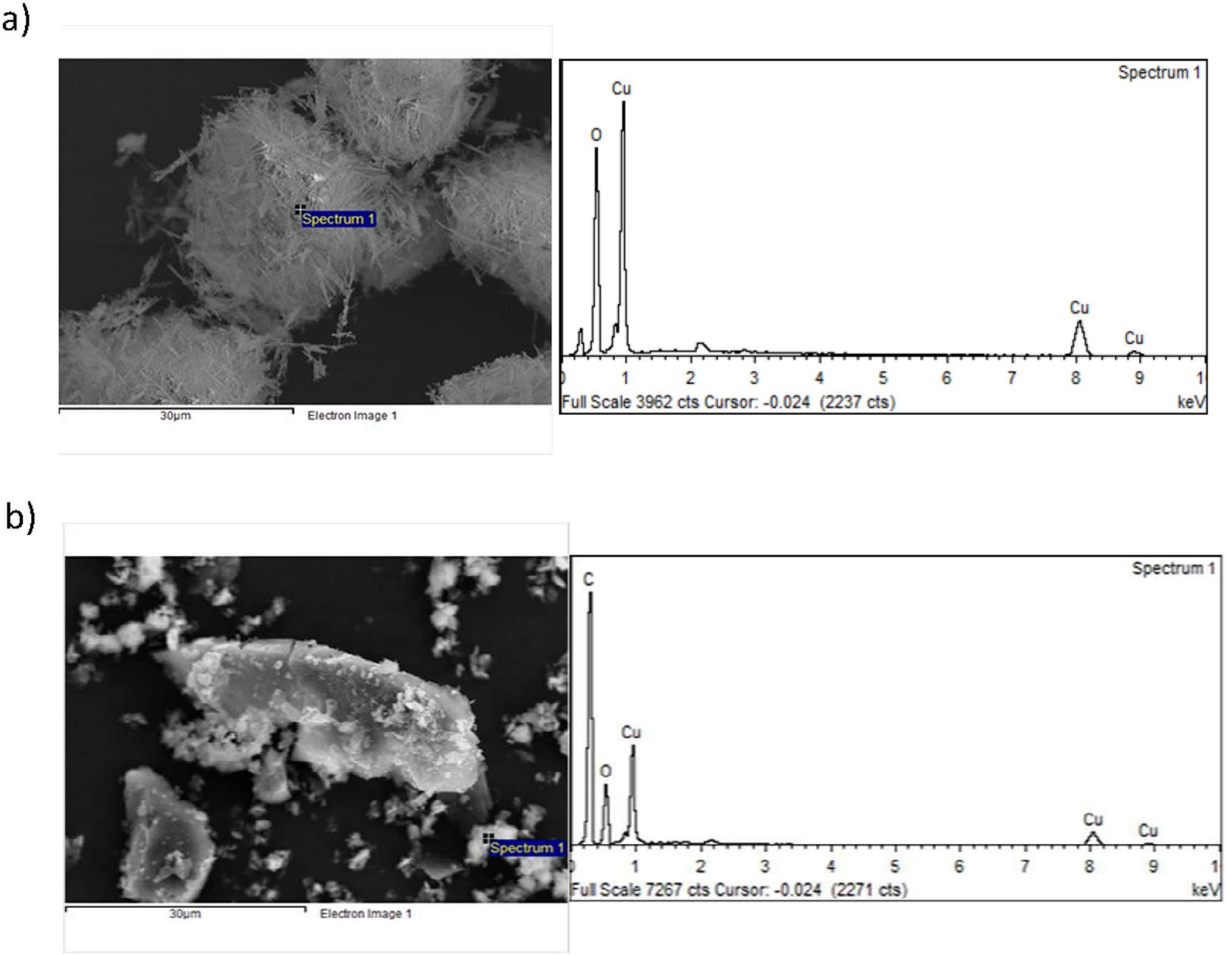
(**a**) SEM–EDS spectra of the Cu(OH)_2_-NWs, (**b**) SEM–EDS spectra of the Cu(OH)_2_-NWs-PVA-AC Nano-composite.

The SEM–EDS analysis of Cu(OH)_2_-NWs and Cu(OH)_2_-NWs-PVA-AC Nano-composite are presented in Fig. 3a,b. Figure 3a confirmed the notable presence of elementary copper. Figure 3b confirmed the excessive amount of carbon and copper elementals. These EDS results confirms that Cu(OH)_2_-NWs and Cu(OH)_2_-NWs-PVA-AC Nano-composite contain copper, oxygen, carbon and no other elements are observed. The prior results demonstrate that Cu(OH)_2_ is formed as nanowires bundles, and Nano-composite also fabricated with nanowires bundles. These results corroborate with XRD and SEM analysis.

The adsorption–desorption experiment utilizing nitrogen gas (N_2_) was carried out at 77 K. The N_2_ isotherm is used to identify the specific surface area executing the multipoint method of BET. Based on Table 1, the BET surface area identified by Cu(OH)_2_-NWs-PVA-AC was found to be 384 m^2^/g. It should be mentioned that the specific surface areas and pore volume of Cu(OH)_2_-NWs-PVA-AC were appreciably greater than similar adsorbent products which were reported by previous researchers in the literature. The high specific surface area of this adsorbent can be attributed to enhancing in inter-particle of pore volume^23^.

**Table 1:** Textural characteristics of Cu(OH)_2_-NWs-PVA-AC nano-composite.

Specification	Unit
BET_surface area_	384 m^2^/g
Langmuir_surface area_	569 m^2^/g
BJH_surface area_	167 m^2^/g
Pore volume	0.126 cm^3^/g
Pore size	0.77 nm

**Figure 4:**
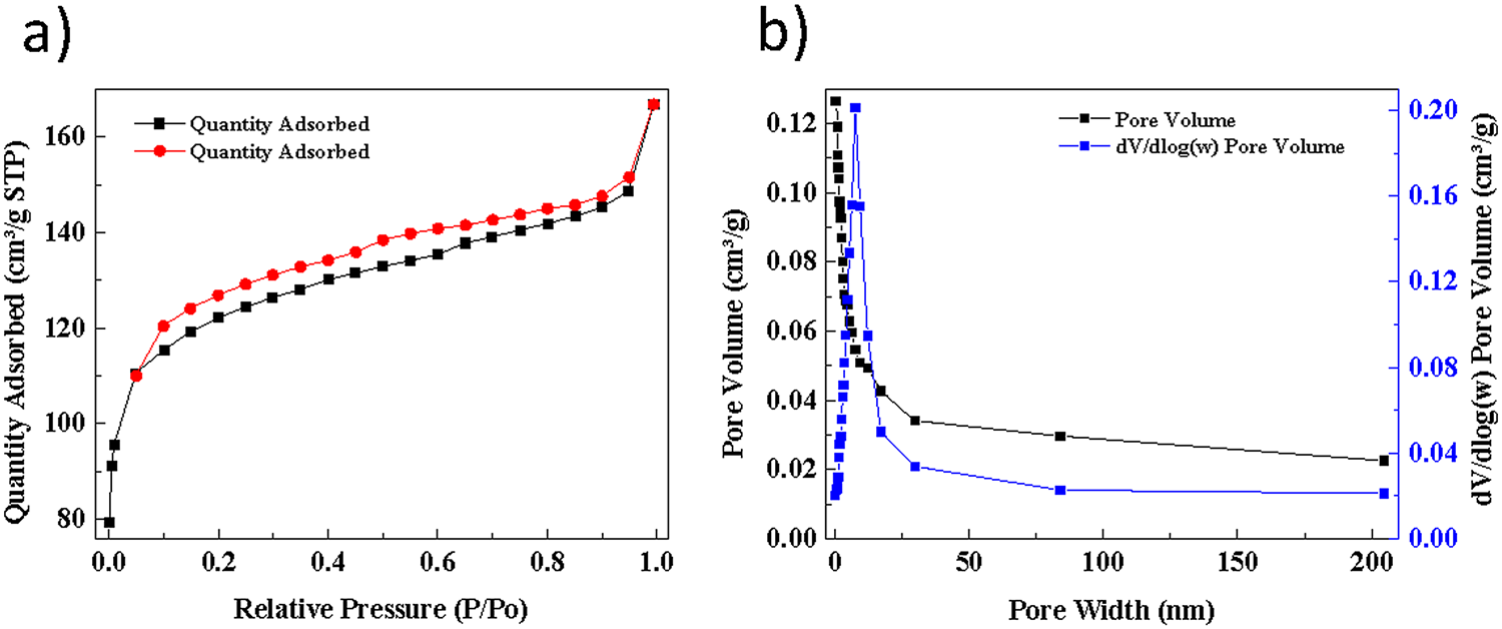
(**a**) N_2_ adsorption/desorption isotherms. (**b**) Pore size distribution and pore volume of raw Cu(OH)_2_-NWs-PVA-AC.

Figure 4a,b show the adsorption/desorption isotherm. According to the classification of IUPAC, the type II isotherm with type H3 hysteresis loop (Fig. 4a). The typical isotherm with H3 hysteresis represents the mesoporous materials with a wide size distribution which has an average width and volume of its pores determining 0.77 nm and 0.126 cm^3^/g, respectively as illustrated in Fig. 4b and the type-II of isotherm is forming multilayer adsorption, and generally, with H3 type hysteresis loop is forming a cluster and platy particles aggregate^24^.

**Figure 5:**
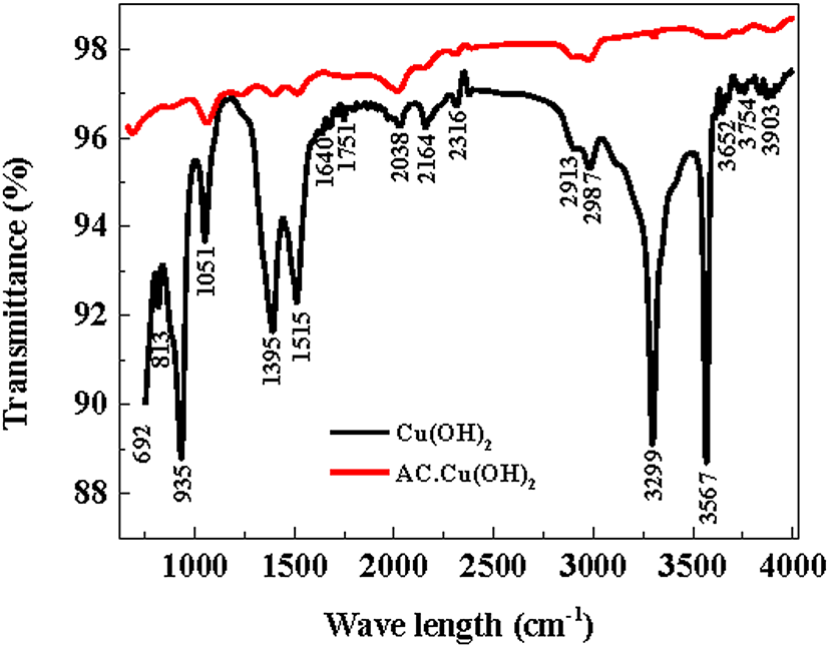
FTIR patterns of Cu(OH)_2_-NWs, and Cu(OH)_2_-NWs-PVA-AC Nano-composite.

Figure 5a,b shows FTIR patterns of Cu(OH)_2_-NWs and Cu(OH)_2_-NWs-PVA-AC Nano-composite respectively. According to the bending mode of peaks presence at 3299‒3567 cm^−1^ has supported the formation of hydroxyl groups in the Cu(OH)_2_-NWs^25^. The band around 1395‒1515 cm^−1^ shows the stretching mode for the absorbed water inside the Cu(OH)_2_-NWs^26^. The C‒O stretching peak around 813‒935 cm^−1^ can be attributed to the relation of the metal cation (Cu^2+^) in Cu(OH)_2_-NWs-PVA-AC Nano composite^27^. The band between 935 and 1051 cm^−1^ in the Nano-composite material is resulting from NWs-PVA-AC^23^. A broad peak around 3046‒3565 cm^−1^ in Cu(OH)_2_-NWs-PVA-AC Nano-composite is found instead of two separate peaks that existed in the Cu(OH)_2_-NWs and can be attributed to incomplete elimination of hydrogen bonds^27^.

On comparison, the FTIR of spectra of the Cu(OH)_2_-NWs-PVA-AC Nano-composite after adsorption with the MB dye, the surface functional groups had roughly changes (Fig. S3, Supplementary Information). As illustrated in Fig. S3, the peak assigned from 3000 to 3500 cm^−1^ can be ascribed to the O‒H group. The vibration band at 1629 cm^−1^ represents the C=C stretching vibration of aromatic benzene rings present in the MB^27^. Cu(OH)_2_-NWs-PVA-AC Nano-composite had interactions with the π e^−^s of the benzene ring present in the MB dye. Consequently, the bending vibrations of the ‒OH group and stretching vibration of C‒OH is the distinguishing band around 1000–1500 cm^−1^^27^. After MB dye adsorption, the distinctive FTIR bands present at 1389 and 1629 cm^−1^ can be accredited to the C–N and C=N bonds, respectively^28^. The existence of C–N and C=N bonds confirmed the MB dye was adsorbed on the surface of Cu(OH)_2_-NWs-PVA-AC Nano-composite successfully.

XPS technique was applied to explore the mechanism of MB removal on Cu(OH)_2_-NWs-PVA-AC Nano-composite. The total survey spectra of the Nano-composite before and after adsorption of MB dye was delineated in Fig. S4. It can be observed the elements of Cu(OH)_2_-NWs-PVA-AC Nano-composite changed after the adsorption of MB dye. In addition to the C, Cu, and O elements before adsorption of MB, N and S elements also present after the MB adsorption (Fig. S4). Thus, we can assume that N and S elements present in MB might be adsorbed successfully on the surface of Cu(OH)_2_-NWs-PVA-AC Nano-composite.

The Carbon 1s and Oxygen 1s spectral analysis before MB dye adsorption were shown in Fig. S5 (Supplementary Information). The C 1s spectra before MB adsorption has shown the main functional groups in Cu(OH)_2_-NWs-PVA-AC Nano-composite. The B.E. values at 284.6, 286.1, and 287.8 eV were caused by sp^3^ C, –C–O and –C=O respectively^27,28^. The O 1s spectra indicate the presence carbons containing species with oxygen functional groups in the Nano-composite. As shown in Fig. S5, the O 1s XPS reveals that the B.E. value of 529.6 eV was corresponding to the O^2−^ ions present in the CuO^28^. Likewise, the B.E. values at 532.1 533.6, and 535.7 eV matched to the –O–C=O, –C=O and –O–C–O groups on the surface of Nano-composite^29,30^.

The Carbon 1s and Oxygen 1s spectral analysis after the adsorption MB dye were shown in Fig. S6 (Supplementary Information). The C 1s spectra after MB adsorption has shown the main functional groups in MB dye and Cu(OH)_2_-NWs-PVA-AC Nano-composite. The B.E. values at 285.1, 288.2, and 290.1 eV are due to the –O–H/–C–O, –C=O and –O–C=O respectively. The O 1s spectra indicate important functional groups in the MB dye and the other C–O functional groups in the Nano-composite. As shown in Fig. S6, the O 1s XPS reveals that the B.E. value of 528.9 and 534.1 eV were corresponded to the N–O and S–O bonds present in the MB dye molecule^31^.

Additionally, compared with the O 1s XPS before MB adsorption the peak intensity of –O–C–O groups reduced after MB adsorption, which can be described as the O^2−^ ions in Cu(OH)_2_-NWs-PVA-AC Nano-composite and cationic MB have electrostatic interaction. The influence of Cu(OH)_2_ NWs on the elimination of MB is presented in Fig. S7 (Supplementary Information). As shown in Fig. S7a, the XPS of Cu 2p before adsorption display distinct peaks at B.E. values of 934.6 and 954.4 eV represents the Cu 2p_3/2_ and Cu 2p_1/2_ respectively^32^. These characteristic XPS peaks confirm the presence of Cu^2+^ species in the form of Cu(OH)_2_ on the surface of Cu(OH)_2_-NWs-PVA-AC Nano-composite. The results are in accordance with the powder XRD analysis (Fig. 1). In addition, the satellite peaks corresponding to Cu 2p_3/2_ and Cu 2p_1/2_ appeared at 943.6 and 963.4 eV respectively further authenticate the existence of Cu^2+^ ions on the adsorbent surface^33,34^. The Cu 2p core level XPS after MB dye adsorption was presented in Fig. S7b, which shows that changes in the patterns of XPS peaks with a slight change in the B.E. values.

### Proposed mechanisms of MB dye adsorption

Adsorption mechanism is a significant assignment to explore in the present study of sorption processes. The earlier reports revealed that some possible interactions that occurred between the MB and Cu(OH)_2_-NWs-PVA-AC are accountable for the adsorption, like electrostatic interactions, hydrogen bonds, and electron donor–acceptor interactions. In addition, the MB dye molecule thickness, depth, and width are equal to 1.43, 0.61, and 0.4 nm, respectively. These dimensions permit the dye to have an easy entry within the porous structure of Cu(OH)_2_-NWs-PVA-AC with a pores size diameter of 0.77 nm (Table 1). Besides, the adsorption process also dependent on the adsorbent functional groups. These surface functional group on top plays a significant role in the adsorption capacity and the elimination mechanism of the MB dye. From the activated carbon the key functional groups of carboxyl and hydroxyl are lending to adsorb cationic MB by electrostatic interaction. Further, the hydrogen bond was responsible for the force between the MB dye and Cu(OH)_2_-NWs-PVA-AC, which contains O, N, and H atoms. The C–C bond could make the potentially stableadsorption system to avoid desorption of MB dye^35^.

The ‒OH and ‒COOH groups present on the surface of Cu(OH)_2_-NWs-PVA-AC (CNPA) had electrostatic interactions under alkaline conditions with cationic dye MB (Eqs. (3), (4)). As evidenced by XPS (Figs. S6, S7, Supplementary Information), the peak area of ‒OH and ‒COOH and the total area of O 1s spectra decreased after reacting with MB. This can beassigned to the reason that ‒OH and ‒COOH on the surface of CNPA bonded with MB as shown in Eqs. (4) and (5) respectively^36^.

3 $${\text{CNPA}}-{\text{OH }} + {\text{ OH}}^{ - } \rightleftharpoons {\text{CNPA}}-{\text{O}}^{ - } + {\text{ H}}_{{2}} {\text{O,}}$$

4 $${\text{CNPA}}-{\text{COOH}} + {\text{ OH}}^{ - } \rightleftharpoons {\text{CNPA}}-{\text{COO}}^{ - } + {\text{ H}}_{{2}} {\text{O,}}$$

5 $${\text{CNPA}}-{{\text{O}}^{-}} \, + {\text{ MB}}^{ + } \rightleftharpoons {\text{CNPA}}-{\text{O}}-{\text{MB,}}$$

6 $${\text{CNPA}}-{{\text{COO}}^{-}} \, + {\text{ MB}}^{ + } \rightleftharpoons {\text{CNPA}}-{\text{COO}}-{\text{MB}}.$$

### Effect of p^H^

**Figure 6:**
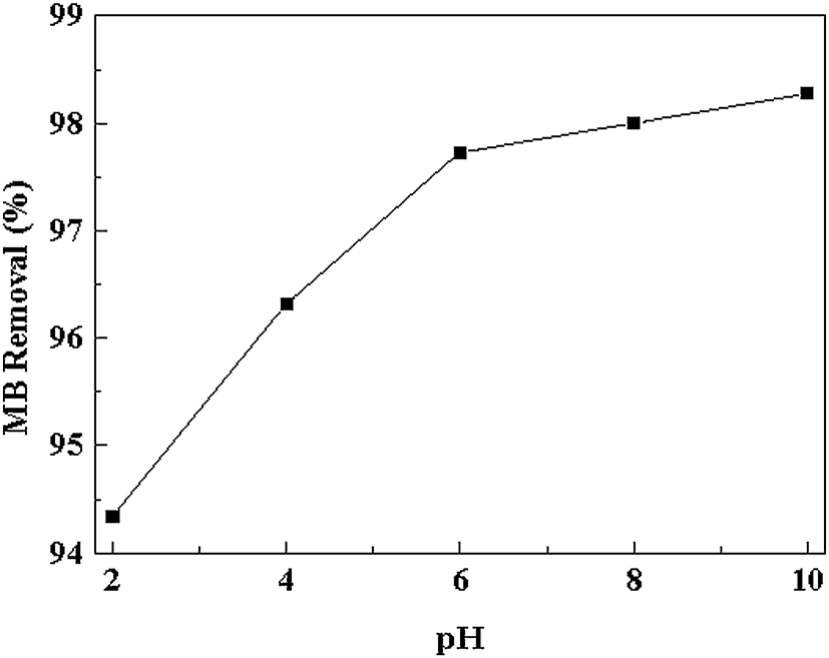
Effect of p^H^. Conditions: MB concentration: 50 mg/L; Volume = 25 mL, Absorbent dose = 30 mg; Temp. = 30 ± 10 °C; Speed of agitation = 200 rpm; Contact time = 60 min.

The influence of solution p^H^ is a significant control parameter in the adsorption process. MB being a cationic dye, it has a positively charged species and p^H^ is 7–8. We attain the accurate study of the MB dye adsorption onto Cu (OH)_2_-NWs-PVA-AC with different p^H^ ranging between 2 and 10 and the results are shown in Fig. 6. The present study shows that a gradual raise in the p^H^ from 2 to 10 results increase in the adsorption capacity. The surface of adsorbent becomes positively charged at lower p^H^ due to protonation of hydroxyl ions present in PVA and Cu(OH)_2_; thereby, cationic MB dye leads to a strong electrostatic repulsion effect between adsorbent and MB dye^18^. Furthermore, as the solution p^H^ decreases, more H^+^ ions are encountered with the positively charged MB and covering the active sites of the Cu(OH)_2_-NWs-PVA-AC surface. At a higher p^H^ level, more binding sites are free and there is less competition between the H^+^ ions and the cationic MB dye^6^. Moreover, all O‒H groups are free and increasing in number. Thus, it was observed that the reaction at higher p^H^ can render a strong electrostatic attraction against MB. These results concluded that MB dye adsorption on Cu(OH)_2_-NWs-PVA-AC is p^H^-dependent. Similar results were attained and disclosed by other researchers^37,38^.

### Effect of contact time

**Figure 7:**
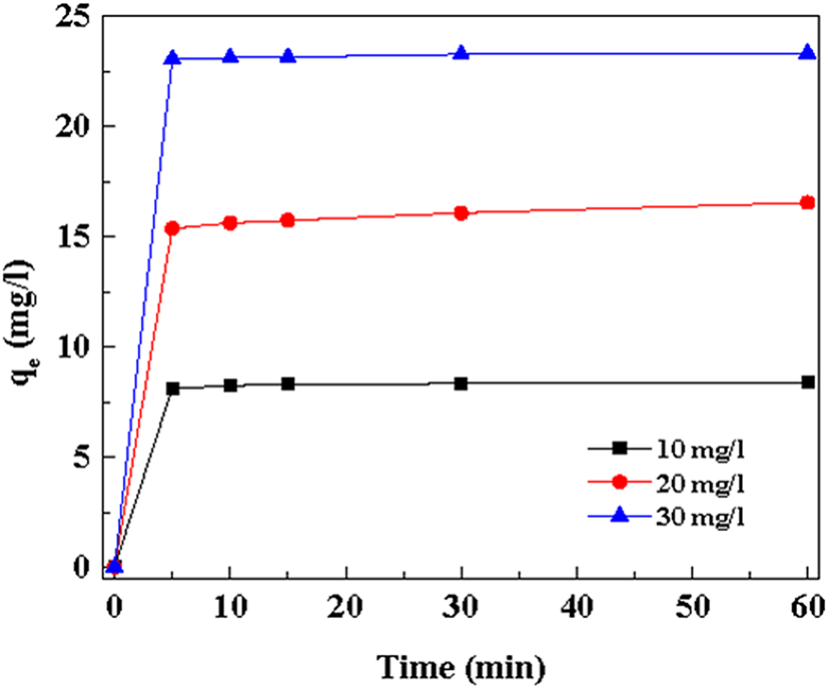
Effect of dye concentration and contact time on MB. Conditions: MB concentration: 10, 20 and 30 mg/L; Volume = 25 mL, Absorbent dose = 30 mg; Temp. = 30 ± 10 °C; Speed of agitation = 200 rpm; Contact time = 60 min.

The adsorption investigation was performed for various contact time intervals (0–60 min). Figure 7 showed the adsorption of MB was enhanced when the contact time was increased to 10 min. In addition, it was observed that an increase in contact time did not show any significant increment in the adsorption processes. At the beginning stage, the rate of adsorption was extremely rapid; after that, the adsorption process was almost moderate. Because the adsorption of MB dye molecules occurred onto the outside surface of Cu(OH)_2_-NWs-PVA-AC initially and then the MB dye molecules slowly enter the internal surface of the pores. In the initial stages, adsorption is faster because of the existence of an extensive number of binding sites for adsorption. At the ending stage, the adsorption processes found very slow because of the saturation of the binding sites which leads to occur the equilibrium^39,40^. Generally, high adsorption capacity materials providing a high surface area (384 m^2^/g) and more adsorption sites with short adsorption equilibrium^41^. This phenomenon leads to the adsorption of the huge contaminant within a limited time. In this experiment, the adsorption equilibrium was acquired within 10 min. Therefore, Cu(OH)_2_-NWs-PVA-AC Nano-composite possesses more ability to remove the MB dye within a shorter-lived period.

### Effect of initial MB dye concentration

The adsorption experiment was carried out in batch mode, and the results revealed that the initial concentration of MB dye solution plays a crucial role as a driving parameter to overcome the mass transit between the two different phases. It was observed that the solution with lower concentrations of MB molecule contains a number of sites, which makes adsorption easier. From this study it was revealed the adsorption rate was higher at the initial stage. Nevertheless, it was noticed the solution contains more MB dye concentration that affects the adsorption capacity due to the saturation of the sites convenient for sorption on the adsorbent.

### Adsorption kinetics

**Figure 8:**
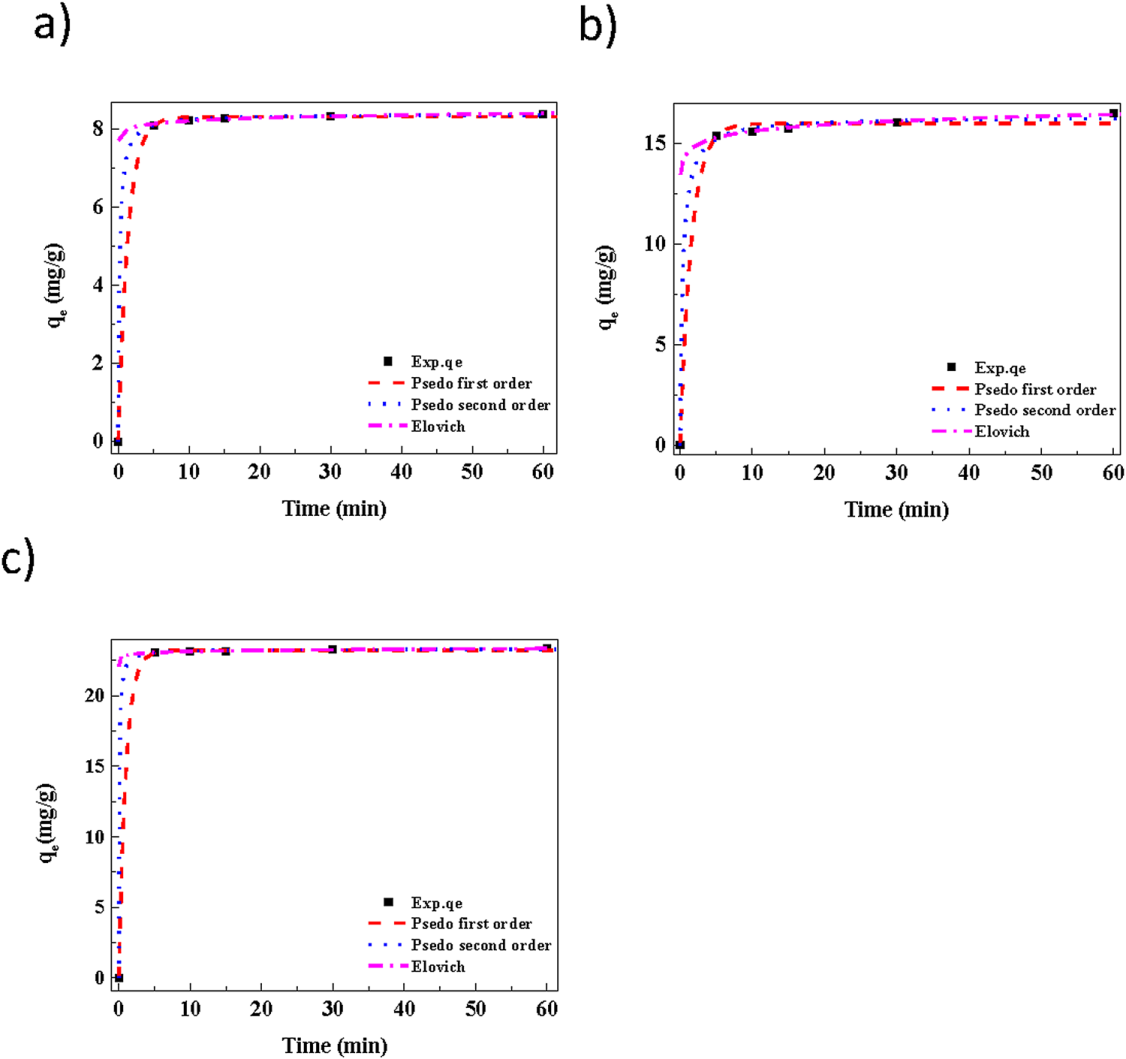
Pseudo-first-order, Pseudo-second-order, and Elovich, models kinetics plots for different concentration: (**a**) 10 mg/L, (**b**) 20 mg/L, (**c**) 30 mg/L. Conditions: As shown in Fig. 7.

Generally, in adsorption study, investigating the kinetic parameter plays a vital role, because it provides information about the mass transfer of molecules/ions from the liquid phase to the adsorbent’s surface. In addition, it also gives the knowledge to understand the adsorption mechanism of MB molecules onto Cu (OH)_2_-NWs-PVA-AC. Figure 8 shows that the adsorption kinetics at various initial MB concentrations. The kinetic data (experimental) including PFO, PSO, Elovich and intra-particle diffusion models are studied^42^. From these models, we can evaluate the kinetic data for MB adsorption and find a reliable model for expressing the experimental q_e_ value. The kinetic equations are shown below:

The PFO model:

7 $$q_{t} = q_{e} \left( {1 - \exp \left( { - K_{1} t} \right)} \right),$$

where the nonlinear form of PSO is

8 $$q_{t} { } = \frac{{K_{2} q_{e }^{2} t}}{{\left( {1 + {\text{q}}_{e} K_{o} t} \right)}}.$$

K_2_ represents the PSO constant (g/mg h), ‘t’ for time (h), q_e_ and q_t_ signify the quantity of MB adsorbed (mg/g) on the surface of Cu(OH)_2_-NWs-PVA-AC Nano composite at equilibrium and at time ‘t’ (h), respectively.

The Elovich kinetic model can be written as,

9 $$q_{t} = \left( {\frac{1}{\beta }} \right)\ln \left( {\alpha \beta } \right) + \left( {\frac{1}{\beta }} \right)\ln \left( t \right),$$

where ‘a’ (g/mg) and ‘b’ (g/mg) are the parameters of the Elovich rate equation.

The PFO, PSO, and Elovich kinetic model data for MB was shown in Fig. 8a–c, respectively. The kinetic parameters data was shown in Table 2. From the R^2^ values, the experimental data is more adopted with the PSO model than the PFO and Elovich models. The R^2^ in PSO model for MB concentrations 10, 20 and 30 mg/L, 0.9999, 0.9991, 0.9999, respectively^43^.

**Table 2:** Estimated kinetic model parameters for methylene blue adsorption on Cu(OH)_2_-NWs-PVA-AC nano composite at different concentration.

Kinetic models	MB-10 (mg/L)	MB-20 (mg/L)	MB-30 (mg/L)
***q***_***t***_** = k**_***2***_***q***_***e***_^**2**^***t/(1 + q***_***e***_***k***_**2**_***t)***			
*q_*e*_	8.3952	16.3484	23.3005
*k*_2_	0.6541	0.1671	0.7671
*R*^2^	0.9999	0.9991	0.9999
*SE*	0.0070	0.2159	0.0479
*NSD*	2.54E^−05^	0.0163	0.0004
*ARE*	2.03E^−05^	− 0.0131	− 0.0003
***q = qe(1−exp(−k***_**1**_***t))***			
*q*_*e*_	8.3148	16.0109	23.2219
*k*_1_	0.7317	0.6371	1.0153
*R*^2^	0.9998	0.9976	0.9999
*SE*	0.0506	0.3529	0.0825
*NSD*	0.0033	0.0459	0.0013
*ARE*	− 0.0026	− 0.0367	− 0.0010
**q_***t***_** = (1/β)ln(αβ) + (1/β)lnt**			
*β*	9.3382	2.1530	8.6743
*α*	2.219E^+31^	1.918E^+13^	1.611E^+85^
*R*^2^	0.9170	0.9726	0.9547
*SE*	0.0356	0.0867	0.0257
*NSD*	0.0012	0.0018	0.0003
*ARE*	− 0.0009	− 0.0014	0.0002

*q; adsorbed MB dye (mg/g) at time t (min).

**The simple Elovich parameters were estimated without using the origin (q = 0, t = 0).

**Figure 9:**
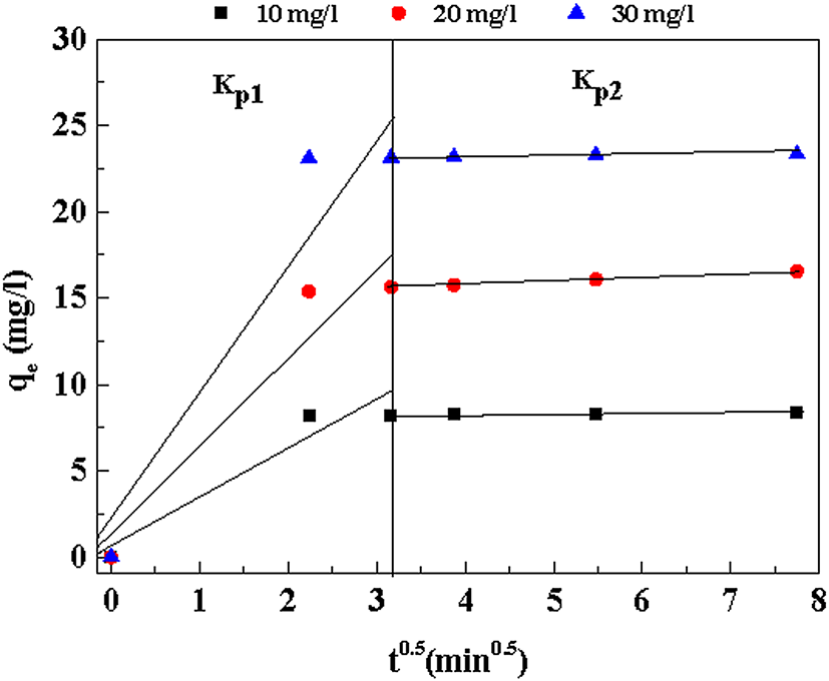
Adsorption Intraparticle diffusion model for adsorption of MB onto Cu(OH)_2_-NWs-PVA-AC Nano-composite. Conditions: As shown in Fig. 7.

The obtained R^2^ values very closer to unity than PFO and Elovich models. Additionally, PSO model calculated equilibrium adsorption capacity values (q_ecal_) also in concurrence with the experimental q_e_ value, as compared with the other kinetic models. According to the result, the adsorption behavior of Cu(OH)_2_-NWs-PVA-AC Nano-composite for MB very well fitted the PSO model with the chemisorption process. For understanding the sorption processes and mechanism of MB by Cu(OH)_2_-NWs-PVA-AC Nano-composite. Weber and Morris intra-particle diffusion model also studied. From Fig. 9, the plot of q_t_ vs t^0.5^ and the data was revels that, all dye concentrations, K_id1_ values are shown to be higher than that of K_id2_ values which shows that an initial period, due to the availability of active sites the dye ions are rapidly occupied, after that the dye ions were starts to migrate towards the Nano-composite pores. The ‘C’ value developed from the intercept, which indicates the boundary layer thickness and if the intercept value is larger, greater will be the boundary layer effect. From Table 3, the values of ‘C’ increases with the increase of initial dye concentration indicating that increasing initial dye concentration promotes the boundary layer effect. Further, the plot straight line is not passing through the origin along with having higher values of parameter ‘C’. This type of behavior indicating the intra-particle diffusion was not rate-limiting reaction, its conformed adsorption by the Cu(OH)_2_-NWs-PVA-AC Nano-composite was mainly regulated by chemisorption.

**Table 3:** Intra-particle diffusion model for different concentration of MB.

Linear portion	Constant	MB-10 (mg/L)	MB-20 (mg/L)	MB-30 (mg/L)
First	KP_1_ (mg/g min^0.5^)	2.802	5.298	7.864
	C_1_ (mg/g)	0.439	0.801	1.242
	R^2^	0.921	0.926	0.920
Second	KP_2_ (mg/g min^0.5^)	0.028	0.204	0.045
	C_2_ (mg/g)	8.165	14.962	22.993
	R^2^	0.940	0.999	0.915

Mostly, at higher correlation coefficient (R^2^) exhibits a better fit for the model. Table 2 shows that the statistical study for the adsorption kinetics and this analysis was carried out using some predictive test tools viz., standard error (SE), average relative error (ARE) and normalization standard deviation (NSD), respectively. From Table 2, the PSO model refers that it was statistically conspicuous depends on higher R^2^ values and lower SE, NSD, and ARE values compared with PFO and Elovich models.

### Adsorption isotherms and thermodynamic study

Adsorption isotherms are authentically necessary for fact-finding the adsorption properties of adsorbents. To determine the temperature effect on MB dye adsorption, the adsorption experiment was carried out at different temperatures (308, 315 and 328 K). In this study, the adsorption data for MB on the Cu(OH)_2_-NWs-PVA-AC was adopted to investigate adsorption behavior by the Freundlich, Langmuir, Langmuir–Freundlich, Temkin, and Redlich-Peterson adsorption isotherm models were studied and adsorption isotherm equation are provided in the Table 4^44,45^.

**Table 4:** Estimated isotherm parameters for MB adsorption on Cu(OH)_2_-NWs-PVA-AC Nano composite at different temperatures.

Temperatures	35 °C	45 °C	55 °C
***q = K***_***f***_*** C***_***e***_^**1/n**^			
*K*_*f*_	39.07	41.03	43.03
1*/n*	0.3654	0.216	0.233
*R*^*2*^	0.999	0.998	0.997
*SE*	1.334	3.102	0.3975
*RMSE*	0.944	2.193	2.229
*χ*^*2*^	0.032	0.169	0.135
***q = K***_***L***_***q***_***m***_***C***_***e***_***/(1 + K***_***L***_***C***_***e***_***)***			
*K*_*L*_ (L/mg)	0.404	0.550	0.806
*qm* (mg/L)	107.6	82.18	79.17
*R*^2^	0.997	0.999	0.998
*SE*	1.047	0.149	0.625
*RMSE*	0.741	0.105	0.442
*χ*^2^	0.019	0.0004	0.006
*R*_*L*1_	0.018–0.029	0.2813–0.1902	0.012–0.020
***q = K***_***L***_***q***_***m***_***C***_***e***_^***1/n***^***/(1 + K***_***L***_***C***_***e***_^**1/n**^***)***			
*K*_*L*_ (L/mg)^1*/n*^	0.331	0.473	0.734
*q*_*m*_ (mg/L)	139.9	115.6	85.21
1*/n*	0.710	0.485	0.820
*R*^2^	0.996	0.984	0.988
*SE*	……	……	……
*RMSE*	0.837	1.506	0.981
χ^2^	0.025	0.078	0.036
*R*_*L*2_	0.103–0.142	0.186–0.225	0.030–0.045
***q = A + B Ln(C***_***e***_***)***			
*K*_*t*_ (L/mg)	31.74	36.58	39.48
*B*	24.57	13.68	14.75
*R*^2^	0.996	0.983	0.948
*SE*	1.152	2.256	2.895
*RMSE*	0.815	1.595	2.047
χ^2^	0.023	0.078	0.114

**Figure 10:**
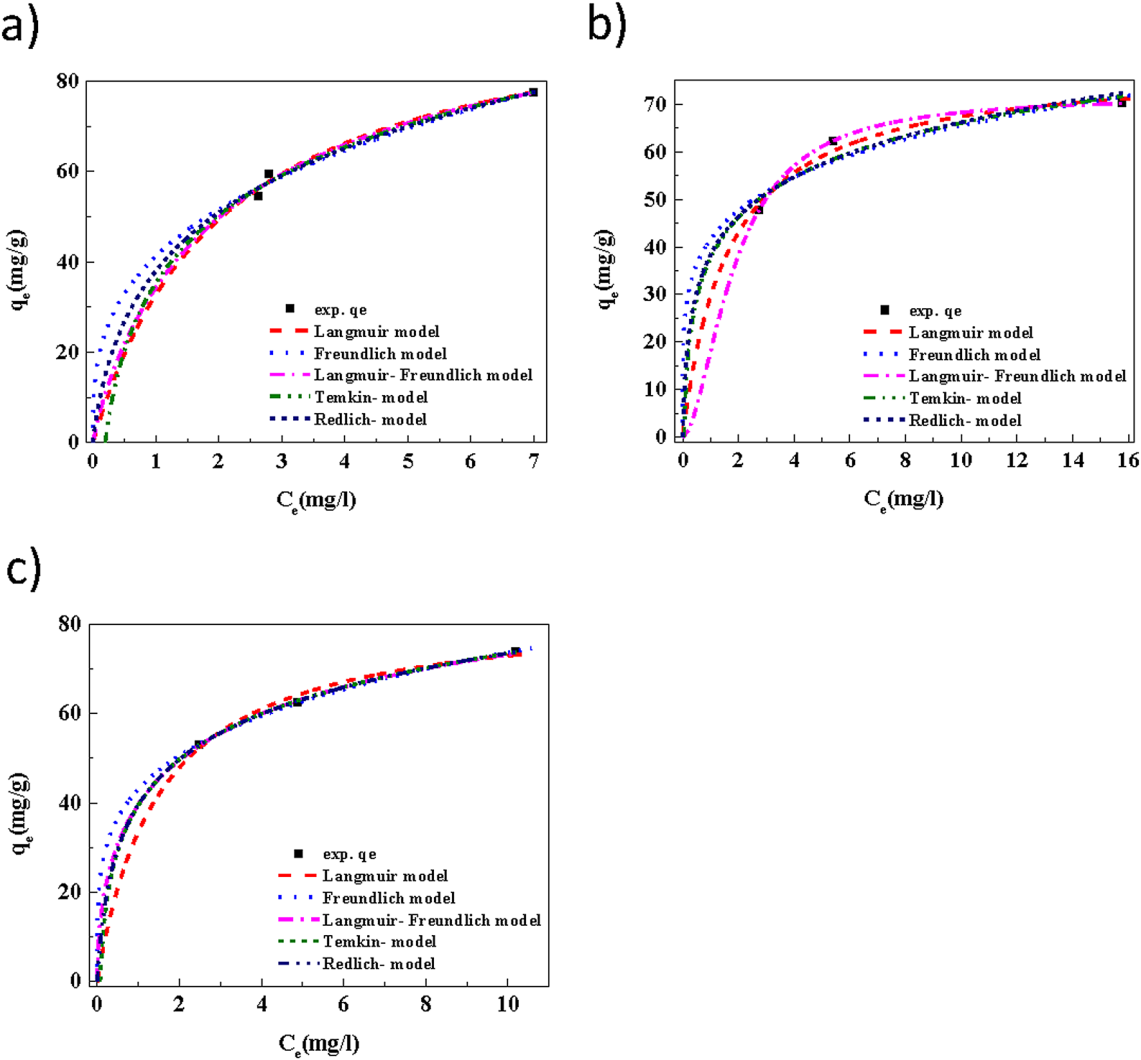
Langmuir, Freundlich, Langmuir–Freundlich, Temkin, and Redlich adsorption isotherms at different temperatures (**a**) 35 °C, (**b**) 45 °C and (**c**) 55 °C) for the adsorption of MB onto Cu(OH)_2_-NWs-PVA-AC Nano-composite. Conditions: MB concentration: 60, 80 and 100 mg/L; Volume = 25 mL, Absorbent dose = 30 mg; Temp. = 35, 45, and 55 °C; Speed of agitation = 200 rpm; Contact time = 60 min.

The adsorption isotherms constant was predicted using the experimental data obtained from nonlinear regression through excel-solver software. Adsorption isotherm non-linear fitting results are illustrated in Fig. 10a–c and fitting parameters are highlighted in Table 4. From the Fig. 10a–c shows the Freundlich, Langmuir, Langmuir–Freundlich, Temkin, and Redlich–Peterson plots are respectively, for adsorption of MB on Cu(OH)_2_-NWs-PVA-AC. From Fig. 10a–c the other parameters are different isotherm constants were calculated, this parameter can be determined by regression of the experimental data. Generally, the two-parameter equation models are Langmuir Freundlich, and Temkin was extensively used than the three-parameter equation models of Redlich–Peterson and Langmuir–Freundlich owing to the troublesomeness of calculating three parameters isotherm model. But, a three-parameter adsorption isotherms models can usually deliver a better fit of the isotherm data than two-parameter models^46^.

The adsorption isotherm data from Fig. 10a–c were evaluated to the above five adsorption isotherm models at different temperatures by using non-linear regression through excel-solver software. The predictable model parameters with the correlation coefficient (R^2^) and standard error (S.E), nonlinear chi-square test (χ^2^) and root mean square error (RMSE) for the different models are tabulated in 4. From the five adsorption isotherm model equations were establish to be statistically notable results. It was established that from among of all models, Langmuir–Freundlich delivered better fitting for the isotherm data in terms of R^2^, SE, RMSE, χ^2^, and RL_1_ values. The Langmuir, Freundlich, and Tempkin equations have fitted the data nearly as well as the three-parameter equations. The Langmuir equation could fine fitting with the adsorption data. Mostly, the applicability of the two-parameter adsorption isotherm models for the present data was roughly following the order: Langmuir–Freundlich > Langmuir > Freundlich > Temkin. In both the Langmuir–Freundlich and Langmuir equations, q_m_ is the amount of the maximum adsorption capacity is 139.9–85.21and 107.6–79.17 mg/g, respectively. From the Langmuir–Freundlich equation at 35 °C, the q_m_was 139.9 mg/g, Langmuir equation q_m_was 107.6 mg/g. The fitting of the adsorption isotherm models are more mathematically meaningful and do not deliver any indication for the definite adsorption mechanisms, the Langmuir–Freundlich and Langmuir models constant can be used for calculating the dimensionless separation factor, which is suggesting of the isotherm shape that predicting the adsorption system favorability. From the Langmuir isotherm, it was found that the monolayer maximum sorption capacity (q_m_) decreased from 107.6 mg/g to 79.17 mg/g with the temperature of the system increasing from 35 to 55 °C, which indicate that adsorption process is exothermic in nature. Generally, the Freundlich model constant (n) value was between 0 and 10, the adsorption process is favorable for chemisorption. Table 4 showed that the n values are apparent in the adsorption process of MB onto Cu(OH)_2_-NWs-PVA-AC Nano-composite was more favorable for chemisorption processes.

The dimensionless equilibrium constant R_L_ also determined for the Langmuir and Langmuir–Freundlich models. It suggests to the possibility of the adsorption process being irreversible (*R*_L_ = 0), favorable (0 < *R*_L_ < 1), linear (*R*_L_ = 1), or unfavorable (*R*_L_ > 1). This separation factor (R_L_) is expressed by the following equation:^47^

10 $$R_{L} { } = \frac{1}{{\left( {1 + {\text{K}}_{L} C_{0} } \right)}},$$

where ‘b’ is the Langmuir constant, while ‘C_0_’ is the initial concentration.

From Table 4, all the R_L_ values within the range of 0 < R_L_ < 1 confirmed that the MB dye ions are more favorably adsorbed on Cu(OH)_2_-NWs-PVA-AC Nano-composite. For the validation and quality of fit obtained by the adsorption isotherms for MB onto Cu(OH)_2_-NWs-PVA-AC Nano-composite adsorbent was applied to the various error functions with the correlation coefficient (R^2^). Nonlinear regression was used based on its merging test to reduce the error distribution between the experimental data and the estimated adsorption isotherms. The data interpretation determines through excel-solver software, standard error (S.E), nonlinear chi-square test (χ^2^) and root mean square error (RMSE) is tabulated 4. The result of the present investigation confirms that at the lowest value of S.E, χ^2^ and RMSE with higher values of R^2^ for Langmuir–Freundlich and Langmuir models in the representing experimental values. This confirms that the Langmuir–Freundlich and Langmuir isotherm models establish the optimal fit to the experimental values.The thermodynamic parameters such as enthalpy change (ΔH^0^), standard Gibbs energy change (ΔG^0^), and entropy change (ΔS^0^) for MB dye adsorption onto the Cu(OH)_2_-NWs-PVA-AC Nano-composite were assessed through following equations.

11 $$\Delta {\text{G}}^{0} = - {\text{RT ln K}}_{{\text{d}}} .$$

The Gibbs free energy change, ΔG^0^, is the fundamental criteria of the spontaneity of a particular process. The standard Gibbs free energy was expressed at different temperatures according to the following Eqs. (12), (13) and (14) respectively^48^.

12 $$\Delta {\text{G}}^\circ = { }\Delta {\text{H}}^\circ - {\text{T}}\Delta {\text{S}}^\circ { }{\text{.}}$$

**Figure 11:**
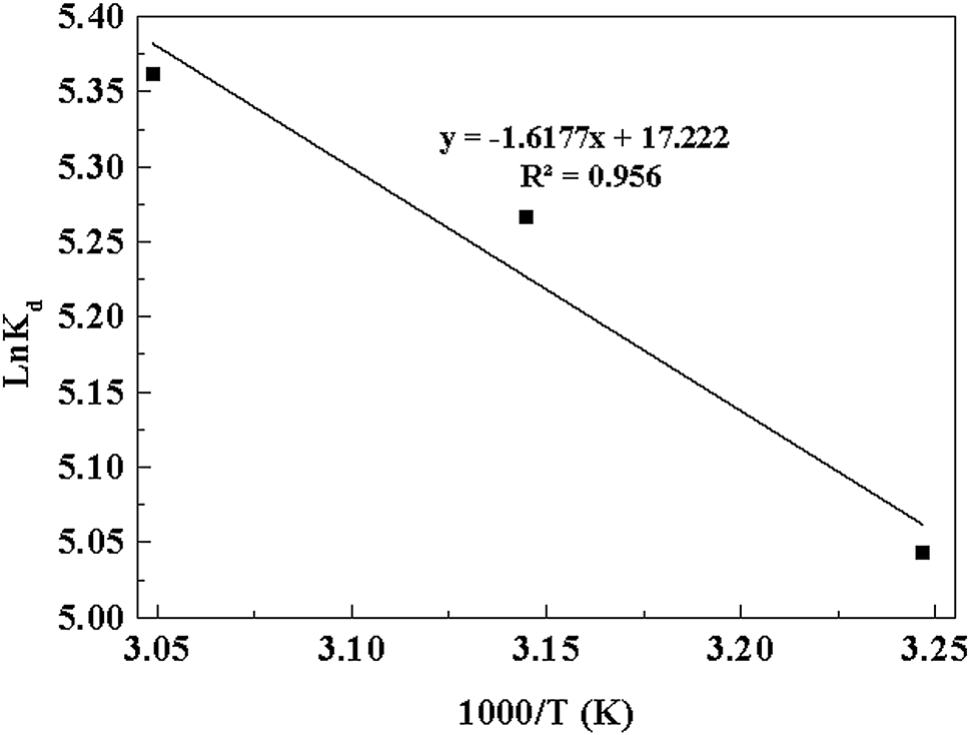
Thermodynamic parameter for MB adsorption onto Cu(OH)_2_-NWs-PVA-AC Nano-composite. Conditions: As shown in Fig. 10.

The enthalpy (ΔH^0^) and entropy (ΔS^0^) change values were calculated from the following equations.

13 $${\text{ln Kd}} = \left( { - \Delta {\text{H}}^\circ { }/{\text{ RT}}} \right) + { }\Delta {\text{S}}^\circ /{\text{R, }}$$

14 $${\text{ln Kd}} = {\text{b }}\left( {{\text{L}}/{\text{g}}} \right){ } \times {\text{ MW }}\left( {{\text{g}}/{\text{mol}}} \right){,}$$

where ‘R’ is the universal gas constant (8.314 × 10^–3^ kJ/mol K), ‘T’ is the absolute temperature (K) and K_d_ is procured by multiplying Langmuir constant ‘b’. The changes in enthalpy (∆H^0^) and entropy (∆S^0^) were calculated from the slope and intercept of the plot of ln K_d_ versus 1/T (Fig. 11). The ΔG° values are calculated from the Eq. 13 at different temperatures. The data is tabulated in Table 5. The negative value of values of ΔG^0^ indicates, the adsorption of MB is a degree of spontaneous process and thermodynamically favorable at various temperatures (308, 318 and 328 K)^49^. The negative values of ΔH^0^ establish the adsorption process is chemisorption and the adsorption reaction are exothermic in nature. The positive values of ΔS^0^ confirm the affinity of Cu(OH)_2_-NWs-PVA-AC for MB and also leads to an increase in the degree of randomness at the solid-solution interface during the process of adsorption.

**Table 5:** Thermodynamic parameters.

T (K)	ΔG° kJ/mol	ΔH kJ/mol	ΔS kJ/mol K
308	− 44.11	− 28.96	0.237
318	− 46.36		
328	− 48.86		

### Comparison of the present study with previous studies

The adsorption of MB from wastewater using various methods and adsorbents has been deliberate by many researchers, while the removal of MB using Cu(OH)_2_-NW-PVA-AC as adsorbent was examined in the current study. The results for MB removal reported in the literature are summarized in Table 6. From Table 6, the Cu(OH)_2_-NWs-PVA-AC adsorbent shows the higher adsorption capacity with a short time and the highest removal percentage than most of the Nano-composite materials. Additionally, it is less toxic than metal oxides and metal oxide Nano composite. From these results, it can be also determined that Cu(OH)_2_-NWs-PVA-AC composite can be deliberated as the promising adsorbent for the removal of MB from wastewater^50–56^.

**Table 6:** Comparison of MB removal by different adsorbents with Cu(OH)_2_-NWs-PVA-AC.

Adsorbent	Contact time (min)	Removal efficiency (%)	The adsorption capacity (mg/g)	References
Fe_3_O_4_NPs	180	71	3.55	^50^
CuO-NP-AC	35	90	10.54	^51^
CuO-NP-AC	4.2	97.58	21.26	^52^
NiS-NP-AC	5.6	96.9	46–52	^53^
Ag-NP-AC	15	98	71.43	^54^
Pd-NP-AC	15	98	75.4	^54^
ZnS:Cu-NP-AC	2.2	99.5	100	^55^
Cu_2_O-NP-AC	60		110	^56^
Cu(OH)_2_-NWs-PVA-AC	10	96–99	139.9	Present study

### Desorption study

The adsorption of MB was first accomplished under a dye concentration of 50 mg/L and the adsorbent dose of 0.03 g. The adsorbent was then collected through filtration and air-dried for the desorption experiments. The desorption experiments were carried out by shaking the MB dye loaded Cu(OH)_2_-NWs-PVA-AC in 25 mL of different desorbing solvents (H_2_O, EtOH, NaOH, and HCl). This experiment was conducted in a shaker at 200 rpm for 60 min, once the reaction was stopped, the solid adsorbent was separated from the solution through filtration and the dye amount into the solution was determined to calculate the removal amount from the Eq. (15).

15 $${\text{Desorption }}\left( \% \right) = \, \left( {{\text{C}}_{{{\text{des}}}} /{\text{C}}_{{{\text{ad}}}} } \right) \times {1}00.$$

*‘C*_des_’ and ‘*C*_ad_’ are respectively the desorbed and adsorbed concentration of the dye.

**Figure 12:**
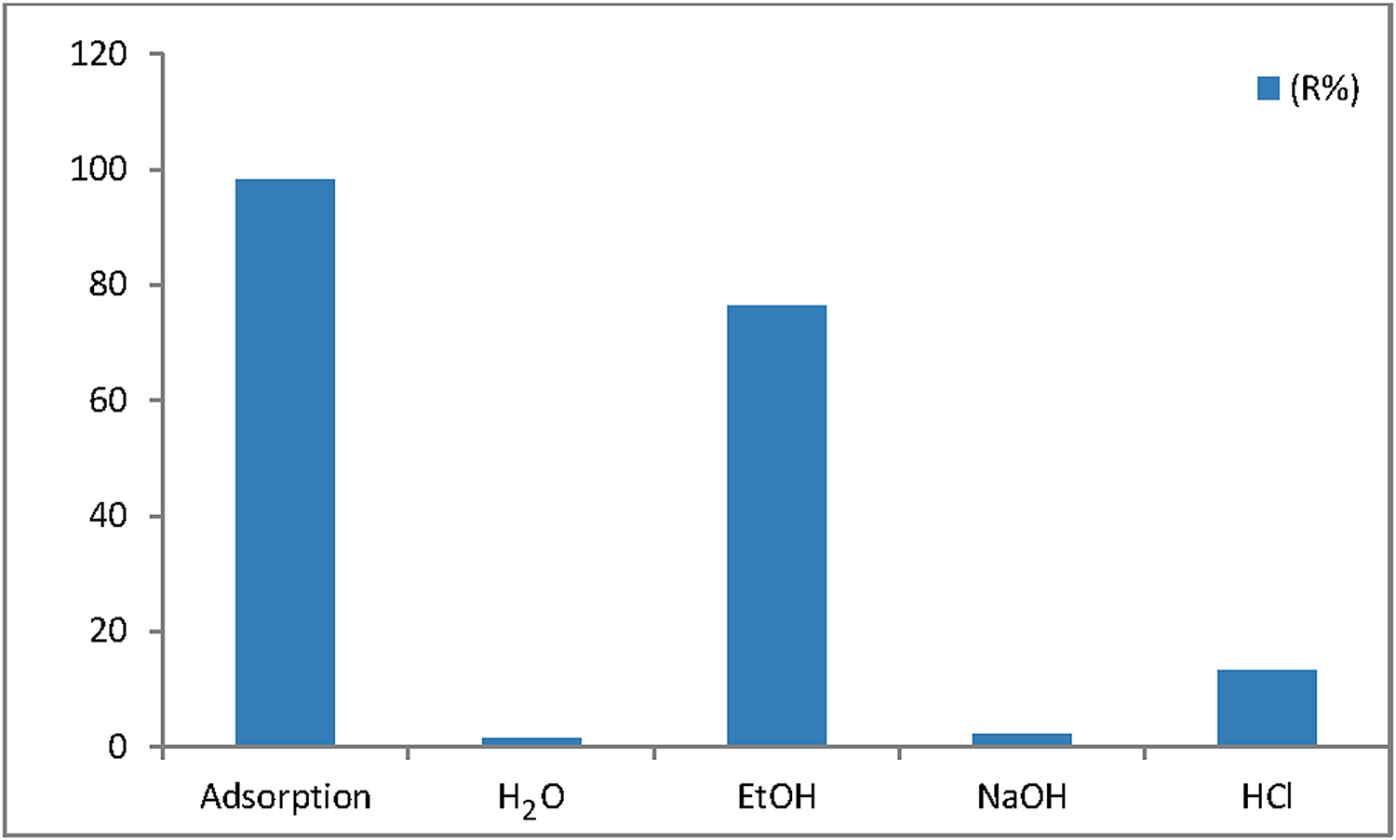
Desorption of MB onto Cu (OH)_2_-NWs-PVA-AC.

Reutilizing of an adsorbent is the most significant way for making it economically viable. The desorption test was performed with different eluents H_2_O, pure EtOH, 0.1 M HCl, and 0.1 M NaOH as designated for the desorption of MB onto Cu (OH)_2_-NWs-PVA-AC and is shown in Fig. 12. Among the four desorbing solvents, EtOH shows the highest MB desorption percentage of 76.47%, and followed by HCl (13.23%). Due, it was conventional a strong electrostatic attraction of MB with the Cu (OH)_2_-NWs-PVA-AC covering and needs higher energy to remove the dye in the chemical regeneration method. Further, the dye desorption efficiency was found to be increased as compared to the other desorbing solvents, because of the dye molecule easily dissolved in the EtOH solvent^56,57^. After EtOH followed by HCl showing the second-highest percentage desorption amount. Due to low adsorption is occurred in acid condition. Knowing the H^+^ from acidic solution easily relocates the Cu(OH)_2_-NWs-PVA-AC ions bonded to the adsorbent during the desorption stage. It will be capable to diffuse and react by the Cu(OH)_2_-NWs-PVA-AC material easily and desorb dye molecules.

## Conclusions

A novel Cu(OH)_2_-NWs-PVA-AC Nano-composite has been prepared by the simple precipitation route at room temperature. The synthesized Nano-composite was employed for the removal of MB from wastewater through a systematic procedure. The adsorption kinetics results exhibit that the adsorption process fits the PSO model with a high correlation value (0.9999), which proposes the rate of adsorption depends on the accessibility of adsorption sites than on dye concentration. The equilibrium data fit the Langmuir–Freundlich and Langmuir isotherm model, demonstrating monolayer coverage of MB molecules over the surface of Cu(OH)_2_-NWs-PVA-AC. The Nano-composite shows 96‒99% of MB adsorption efficiency for a contact time of 10 min. The MB removal capacity exhibits a decline in decreases with a hike in the temperature. Thermodynamic parameters (ΔG°, ΔH°, and ΔS°) show that the chemical adsorption process is exothermic and spontaneous in nature. The results demonstrate that Cu(OH)_2_-NWs-PVA-AC Nano-composite can be regarded as a promising adsorbent for the elimination of MB dye from the aqueous solution. The presence of surface functionality plays a substantial role in the adsorption capacity and the elimination mechanism of the MB dye. Additionally, it is unharmful to the ecosystem and particularly aquatic environment compared to the metal oxide nanoparticles.

## Supplementary Information

Supplementary Information.

## Data Availability

The datasets generated and analyzed during the current study are included in this article and also it is available from the corresponding author on reasonable request.
