# Author Correction: Synthesis and characterization of Cu(OH)_2_-NWs-PVA-AC Nano-composite and its use as an efficient adsorbent for removal of methylene blue

**DOI:** 10.1038/s41598-021-95555-w

**Published:** 2021-08-02

**Authors:** Sivarama Krishna Lakkaboyana, Khantong Soontarapa, Nabel Kalel Asmel, Vinay Kumar, Ravi Kumar Marella, Ali Yuzir, Wan Zuhairi Wan Yaacob

**Affiliations:** 1 Department of Chemical Technology, Faculty of Sciences, Chulalongkorn University, Pathumwan, Bangkok, 10330 Thailand; 2 Center of Excellence on Petrochemical and Materials Technology, Chulalongkorn University, Pathumwan, Bangkok, 10330 Thailand; 3 Building and Construction Technology Engineering, Northern Technical University, 41002 Mosul, Iraq; 4 Department of Biotechnology, Indian Institute of Technology Roorkee, Roorkee, Uttarakhand 247667 India; 5 Department of Chemistry (H & S), PACE Institute of Technology & Sciences, Ongole, Andhra Pradesh 523001 India; 6 Department of Environmental Engineering and Green Technology (EGT), MJIIT-Universiti Teknologi Malaysia, Jalan Sultan Yahya Petra, 54100 Kuala Lumpur, Malaysia; 7 Geology Program, School of Environmental Science and Natural Resources, FST, University Kebangsaan Malaysia, 43600 Bangi, Selangor Malaysia

Correction to: *Scientific Reports* https://doi.org/10.1038/s41598-021-84797-3, published online 11 March 2021

The original version of this Article contained an error in the author name Wan Zuhairi Wan Yaacob which was incorrectly given as Wan Zuhairi Wan Yaacob Zuhairi. The original Article has been corrected.

