# Supplementary material for: Synthesis and characterization of Cu(OH)2-NWs-PVA-AC Nano-composite and its use as an efficient adsorbent for removal of methylene blue
Source: Sci Rep. 2021 Mar 11;11:5686. doi: 10.1038/s41598-021-84797-3 (PMC7970965; doi:10.1038/s41598-021-84797-3)
Supplement: Supplementary file 1 — Supplementary Information. [file 41598_2021_84797_MOESM1_ESM.docx]

**Supplementary Information**

**Synthesis and characterization of Cu(OH)_2_-NWs-PVA-AC Nano-composite and its use as an efficient adsorbent for removal of Methylene Blue**

L. Sivarama Krishna,^1,2*^ K. Soontarapa,^1,2*^ N.K. Asmel,^3^ Vinay Kumar,^4^ Ravi Kumar Marella,^5^ A. Yuzir,^6^ W.Y. Wan Zuhairi^7^

*^1^Department of Chemical Technology, Faculty of Sciences, Chulalongkorn University, Pathumwan, Bangkok, 10330, Thailand.*

*^2^Center of Excellence on Petrochemical and Materials Technology,Chulalongkorn University, Pathumwan, Bangkok, 10330, Thailand.*

*^3^Building and Construction Technology Engineering, Northern Technical University, 41002 Mosul, Iraq.*

*^4^Department of Biotechnology, Indian Institute of Technology Roorkee, Roorkee247667, Uttarakhand, India.*

*^5^Department of Chemistry (H & S), PACE Institute of Technology & Sciences, Ongole 523001, Andhra Pradesh, India.*

*^6^Department of Environmental Engineering and Green Technology (EGT), MJIIT- UniversitiTeknologi Malaysia, Jalan Sultan Yahya Petra, 54100 Kuala Lumpur.*

*^7^Geology program, School of Environmental Science and Natural Resources, FST, University Kebangsaan Malaysia, Bangi-43600, Selangor, Malaysia.*

**Corresponding authors contact information:** L. Sivarama Krishna ([svurams@gmail.com](mailto:svurams@gmail.com)); K. Soontarapa (khantong.s@chula.ac.th).

**
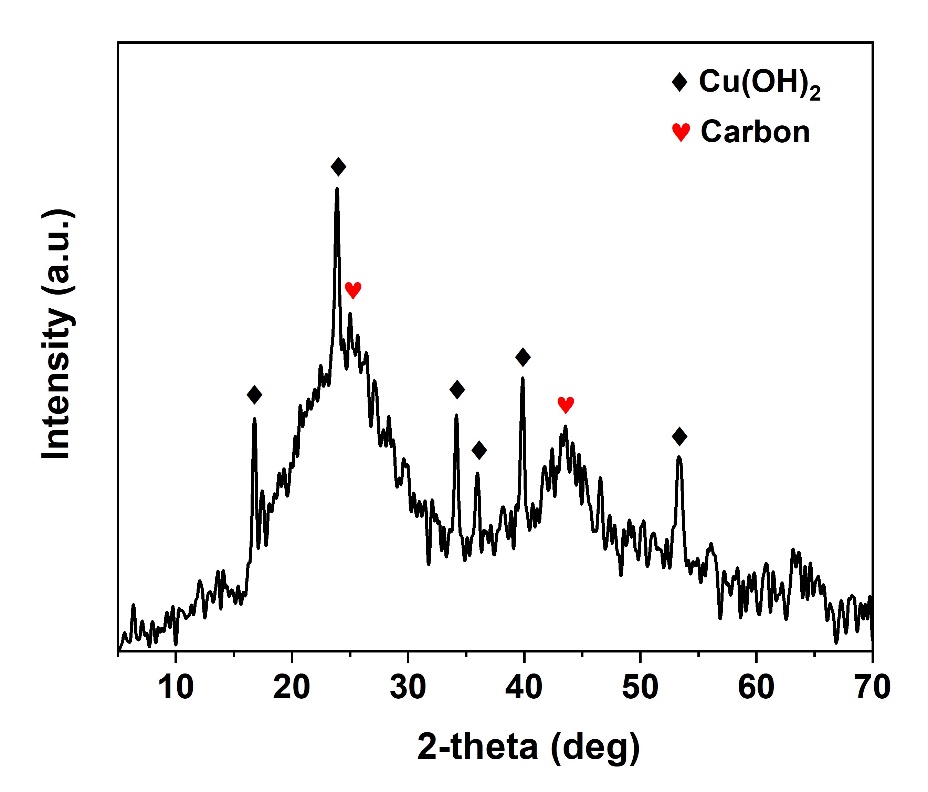
Fig. S1** XRD patterns of Cu(OH)_2_-NWs-PVA-AC Nano-composite after adsorption of MB

**
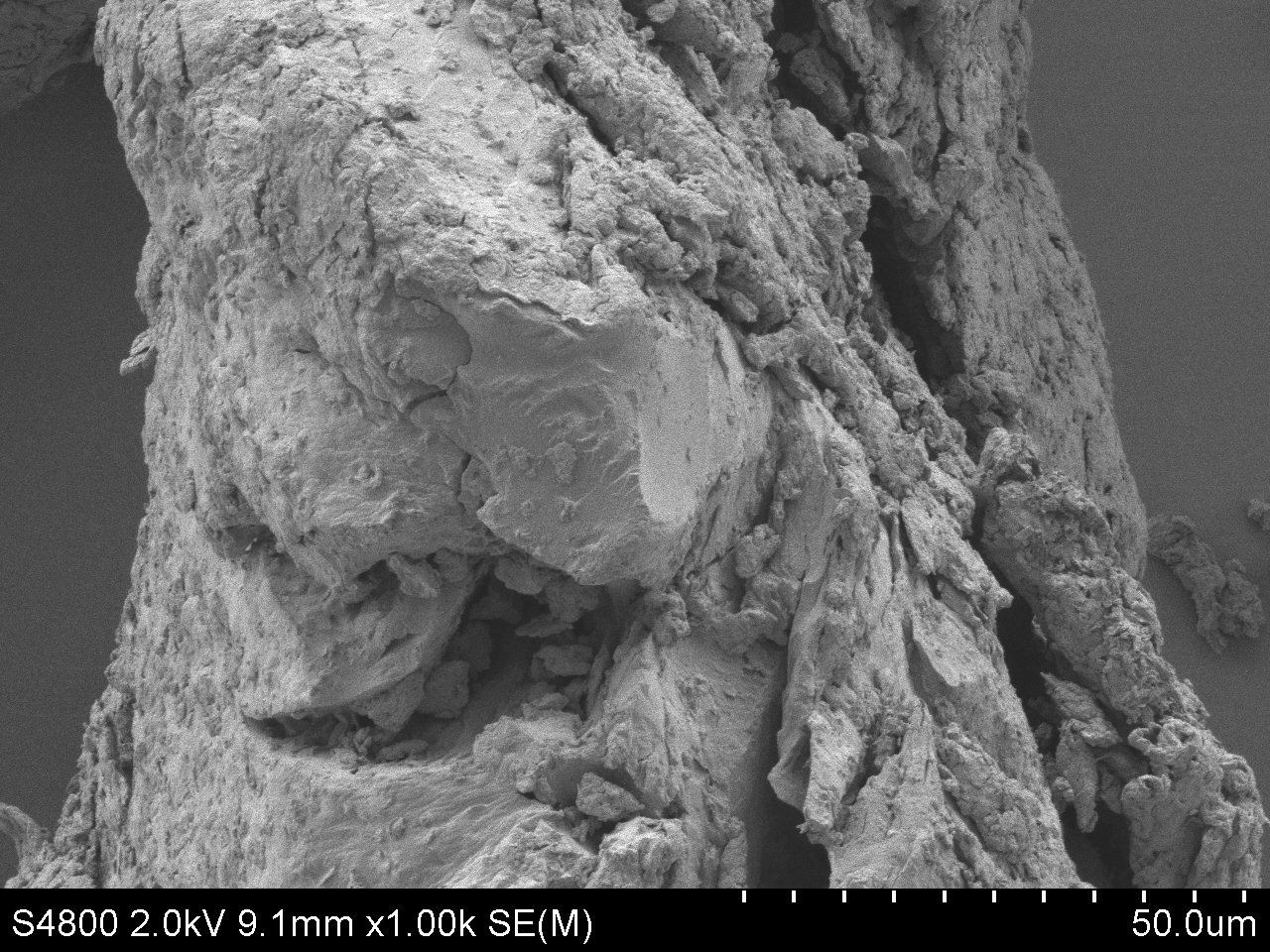
**

**Fig. S2** FE-SEM image of Cu(OH)_2_-NWs-PVA-AC Nano-composite after adsorption of MB

**
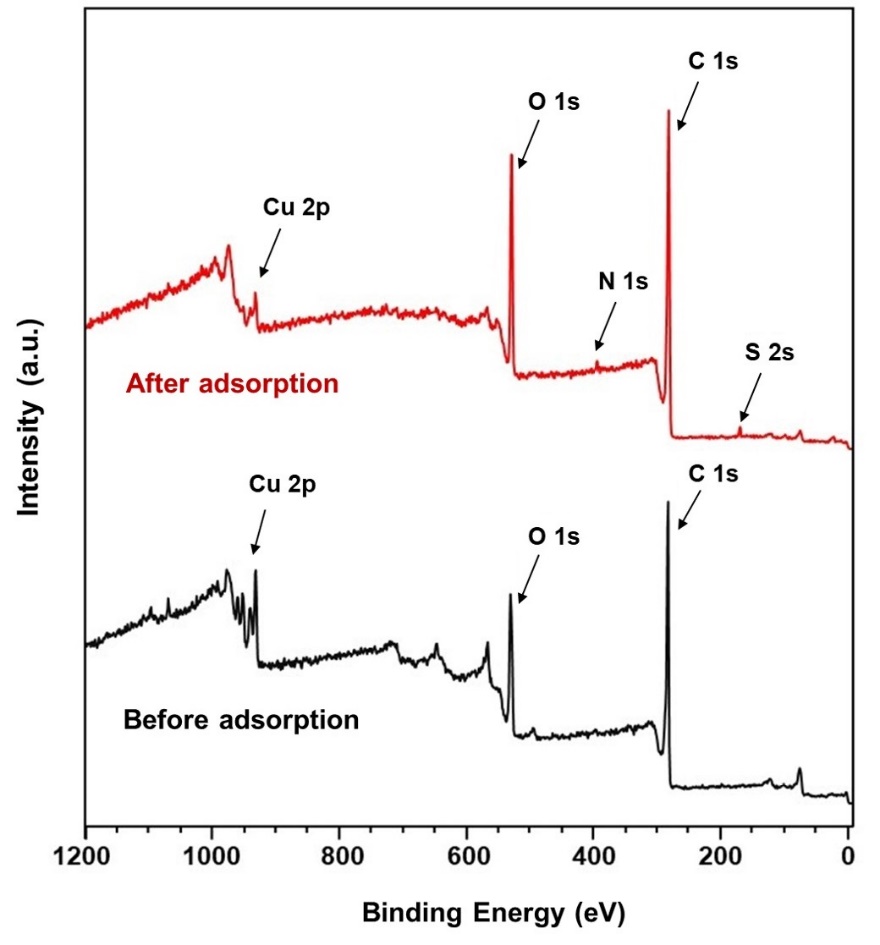

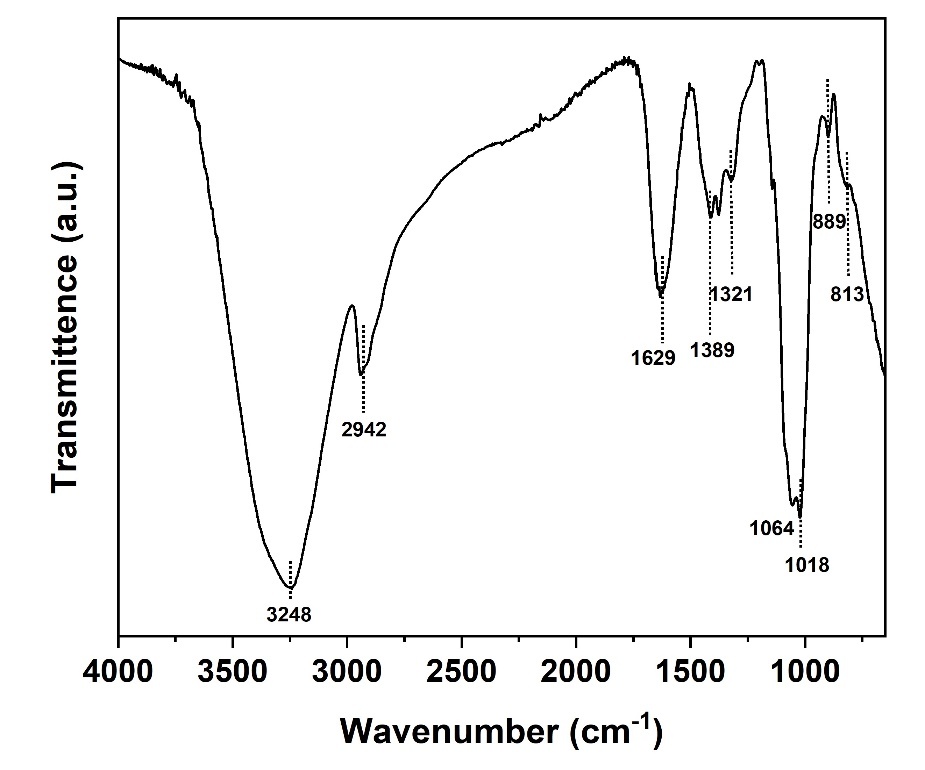
Fig. S3** FTIR spectra of Cu(OH)_2_-NWs-PVA-AC Nano-composite after adsorption of MB

**Fig. S4** XPS total survey spectra of Cu(OH)_2_-NWs-PVA-AC Nano-composite before and after adsorption of MB

**
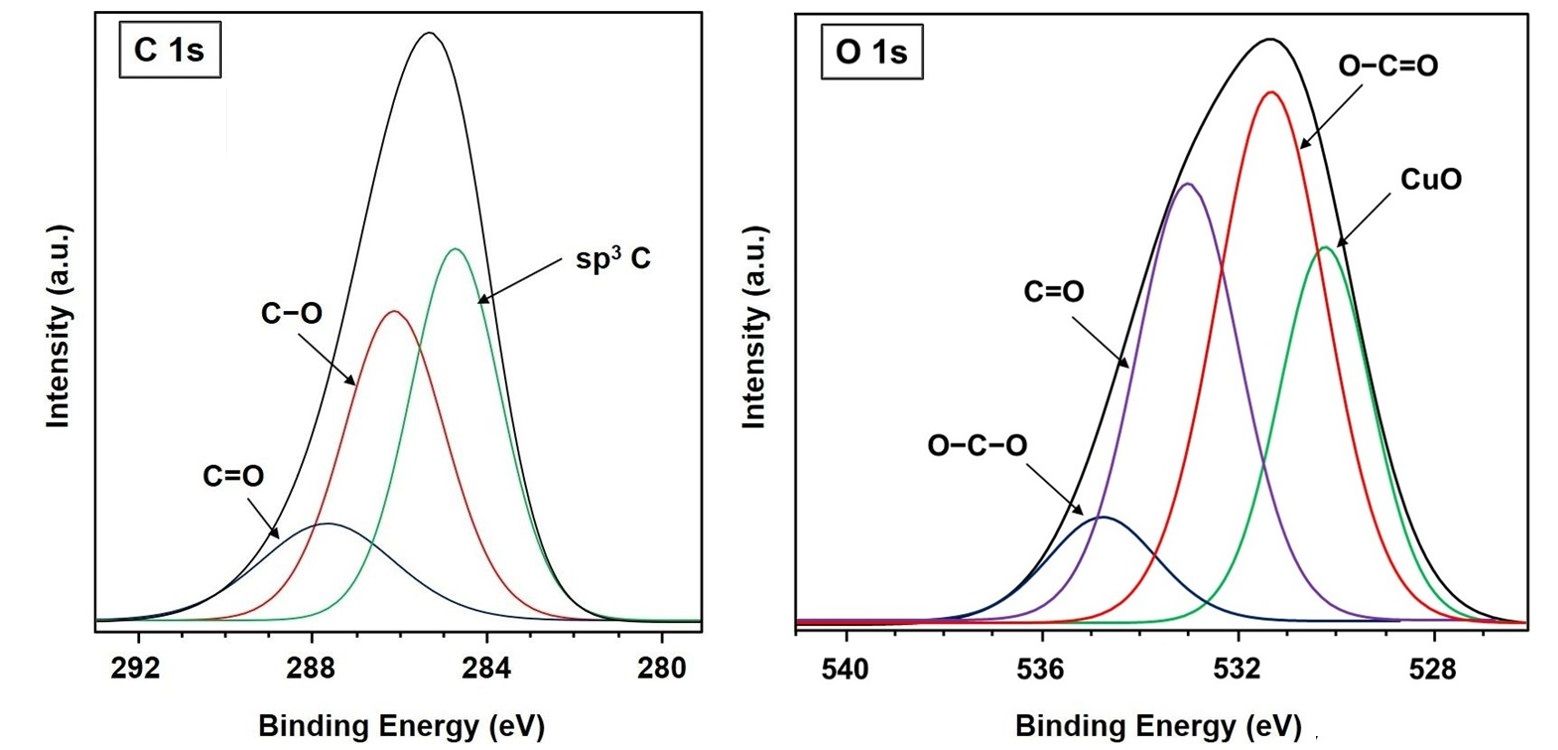
Fig. S5** C1s and O1sspectra of Cu(OH)_2_-NWs-PVA-AC Nano-composite before MBadsorption


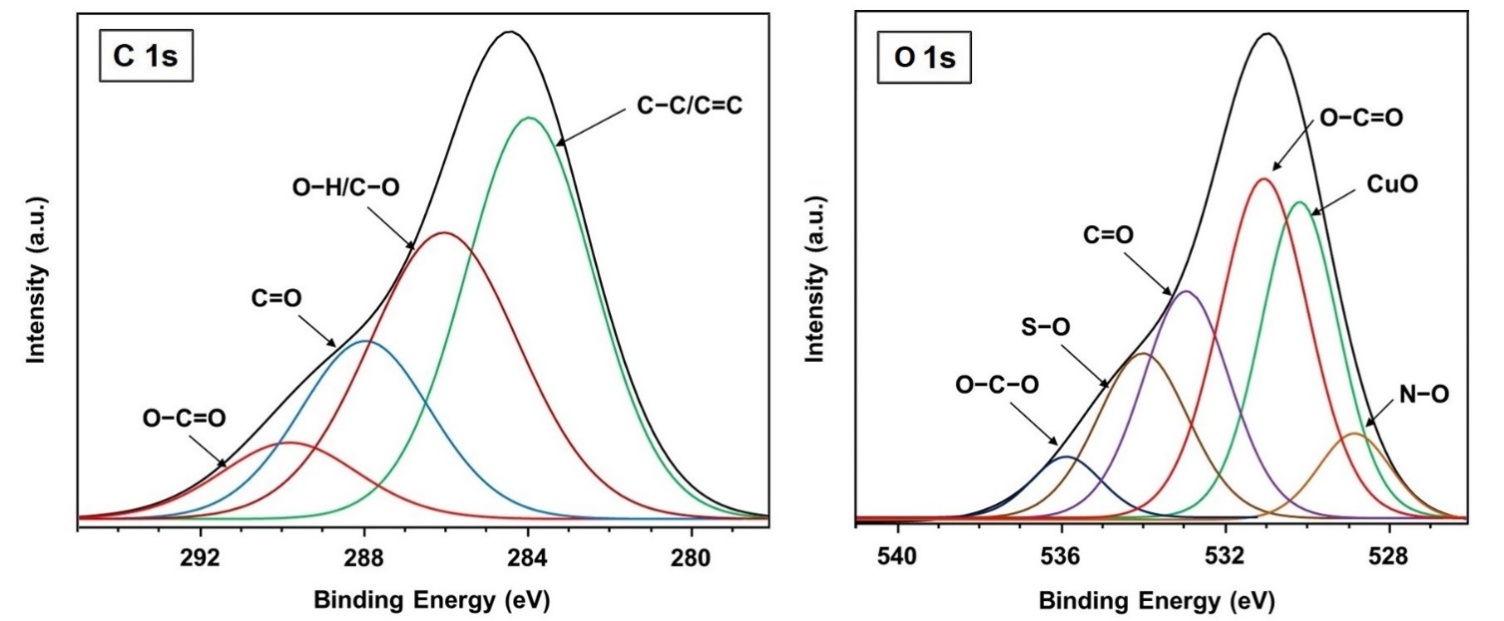


**Fig. S6** C1s and O1sspectra of Cu(OH)_2_-NWs-PVA-AC Nano-composite after MBadsorption

**
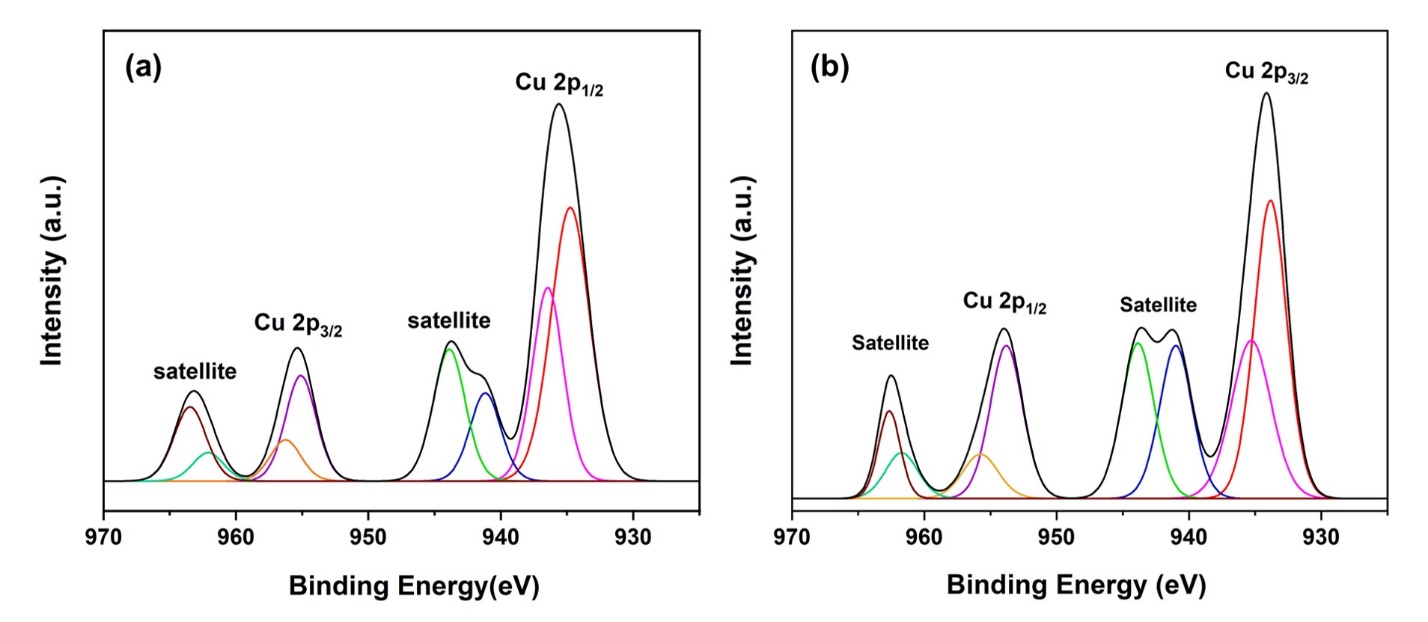
**

**Fig. S7** Cu 2p XPSspectra of Cu(OH)_2_-NWs-PVA-AC Nano-composite (a) before and (b) after MBadsorption
